# Transcriptome reveals insights into biosynthesis of ginseng polysaccharides

**DOI:** 10.1186/s12870-022-03995-x

**Published:** 2022-12-19

**Authors:** Xiaoxue Fang, Huaying Wang, Xinteng Zhou, Jing Zhang, Hongxing Xiao

**Affiliations:** 1 Key Laboratory of Molecular Epigenetics of Ministry of Education, Northeast Normal University, 130024 Changchun, China; 2 Forestry Survey and Design Institute of Jilin Province, 130022 Changchun, China

**Keywords:** Differentially expressed genes, Ginseng, Polysaccharides, Transcriptomes, Transcription factors

## Abstract

**Background:**

Ginseng polysaccharides, have been used to treat various diseases as an important active ingredient. Nevertheless, the biosynthesis of ginseng polysaccharides is poorly understood. To elucidate the biosynthesis mechanism of ginseng polysaccharides, combined the transcriptome analysis and polysaccharides content determination were performed on the roots, stems, and leaves collected from four cultivars of ginseng.

**Results:**

The results indicated that the total contents of nine monosaccharides were highest in the roots. Moreover, the total content of nine monosaccharides in the roots of the four cultivars were different but similar in stems and leaves. Glucose (Glc) was the most component of all monosaccharides. In total, 19 potential enzymes synthesizing of ginseng polysaccharides were identified, and 17 enzymes were significantly associated with polysaccharides content. Among these genes, the expression of phosphoglucomutase (PGM), glucose-6-phosphate isomerase (GPI), UTP–glucose-1-phosphate uridylyltransferase (UGP2), fructokinase (scrK), mannose-1-phosphate guanylyltransferase (GMPP), phosphomannomutase (PMM), UDP-glucose 4-epimerase (GALE), beta-fructofuranosidase (sacA), and sucrose synthase (SUS) were correlated with that of MYB, AP2/ERF, bZIP, and NAC transcription factors (TFs). These TFs may regulate the expression of genes involved in ginseng polysaccharides synthesis.

**Conclusion:**

Our findings could provide insight into a better understanding of the regulatory mechanism of polysaccharides biosynthesis, and would drive progress in genetic improvement and plantation development of ginseng.

**Supplementary Information:**

The online version contains supplementary material available at 10.1186/s12870-022-03995-x.

## Background

*Panax ginseng* C.A. Meyer, which belongs to the Araliaceae, is a well-known traditional Chinese medicine that has been used for several thousands of years. Particularly in China, Korea, and Japan [1]. Ginseng produces several bioactive compounds that are beneficial to human health, including ginsenosides, polysaccharides, proteins, vitamins, alkaloids, and flavonoids [2–4]. Modern pharmacological research showed that ginsenosides was the most effective component. In recent years, research on ginsenosides mainly focused on the pharmacological mechanism of ginseng [5]. At the same time, ginsenosides are not the only effective medicinal ingredients in ginseng. Polysaccharides have attracted more and more attention from medical scientists and nutritionists due to their important biological activity [6]. Since the middle of the 20th century, numerous studies have been conducted on the purification, structure, and biological activity of ginseng polysaccharides. Modern pharmacological studies indicated that ginseng polysaccharides had various biological functions, such as hypoglycemic action, immune-enhancing, and antioxidant activities [7, 8].

Different tissues could produce ginseng polysaccharides, such as roots, leaves, flowers, and berries. Ginseng polysaccharides from these four organs vary in content, structure, and biological activity [9]. Ginseng polysaccharides were biological macromolecules composed of several monosaccharide units linked by glycosidic bonds [10, 11]. Previous studies have shown that the monosaccharide composition contained arabinose (Ara), rhamnose (Rha), galactose (Gal), xylose (Xyl), and glucose (Glc) et al. in ginseng [12, 13]. Most research on ginseng polysaccharides still focuses on the pharmacological activity, nevertheless, the monosaccharide composition and proportion of ginseng polysaccharides are unclear. Moreover, their polysaccharides structures and content were closely related to their biological functions [14–16]. Thus, understanding the composition and proportion of ginseng polysaccharides is great importance to further study the pharmacological activity of ginseng polysaccharides. In addition, despite numerous studies that have been conducted on the structure of ginseng polysaccharides, little is known about the biosynthesis of the major components in ginseng polysaccharides [17].

In China, ginseng has been cultivated for over 1, 000 years [18]. To date, cultivated ginseng generally grouped into eight main types in China, including ‘DaMaYa’, ‘ErMaYa’, ‘YuanBangYuanLu’, ‘ZhuJieLu’, ‘XianLu’, ‘CaoLu’, ‘HuangGuoShen’, and ‘JiShen1’, of which ‘ZhuJieLu’, ‘XianLu’, and ‘CaoLu’ were collectively referred to as ‘ChangBo’ [19]. ‘DaMaYa’ and ‘ErMaYa’ are generally planted in Fusong County, Jilin Province, known as COMMON ginseng. ‘ErMaYa’ and ‘ChangBo’ were commonly cultivated in Ji’an city of Jilin Province, referred to as BIANTIAO ginseng [20]. ‘ChangBo’ and ‘YuanBangYuanLu’ were grown in Kuandian Manchu Autonomous County of Liaoning Province, known as SHIZHU ginseng [19]. Fusong and Ji’an, which account for half of the area of ginseng cultivation in China [21]. COMMON ginseng, BIANTIAO ginseng and SHIZHU ginseng are the three main cultivars of cultivated ginseng in China [20]. GAOLI ginseng was introduced in the Korean Peninsula and was currently cultivated in the Korean Autonomous County of Changbai and Baishan city, Jilin Province, which had developed a new cultivar in China [22]. The difference in morphology, stress resistance, and intrinsic quality have occurred in four ginseng cultivars due to long-term cultivation. The COMMON ginseng exhibits higher cold resistance than other cultivars [19]. In addition to the pharmacological activity of polysaccharides, previous studies showed that increasing sugar content can reduce or avoid cold damage to plants [23]. Is common ginseng’s high cold resistance related to the high content of ginseng polysaccharides? And whether there are differences in polysaccharides content in four ginseng cultivars?

Our study integrated transcriptome sequencing and measurement of the polysaccharides content in the root, stem, and leaf from four ginseng cultivars grown in China. Our specific objectives include (1) investigating the differences in gene expression and polysaccharides accumulation in different ginseng cultivars; (2) inferring the synthetic pathway of ginseng polysaccharides; (3) exploring key regulatory genes and gene regulation network in the biosynthetic pathway of polysaccharides. The results of this work may improve our understanding of the regulatory mechanism and provide new information to develop ginseng plantation for commercial polysaccharides production in China.

## Result

### Polysaccharides accumulation among four ginseng cultivars

**Fig. 1:**
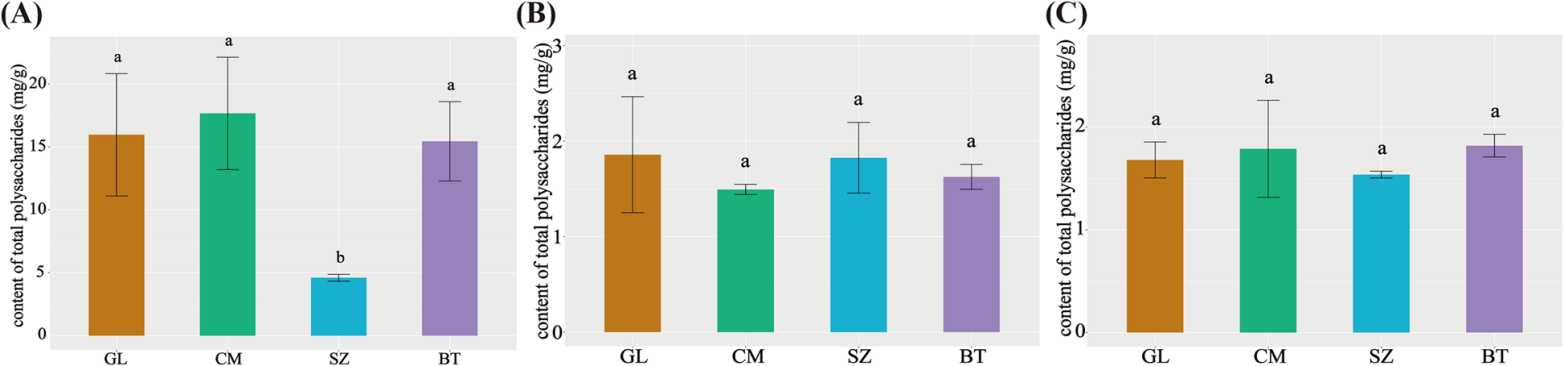
The total content of nine monosaccharides in the (**A**) root, (**B**) stem, and (**C**) leaf of four ginseng cultivars Note: Different lowercase letters indicate significant differences of the total content of nine monosaccharides among the four cultivars in roots, stems, and leaves at the 0.05 level

**Fig. 2:**
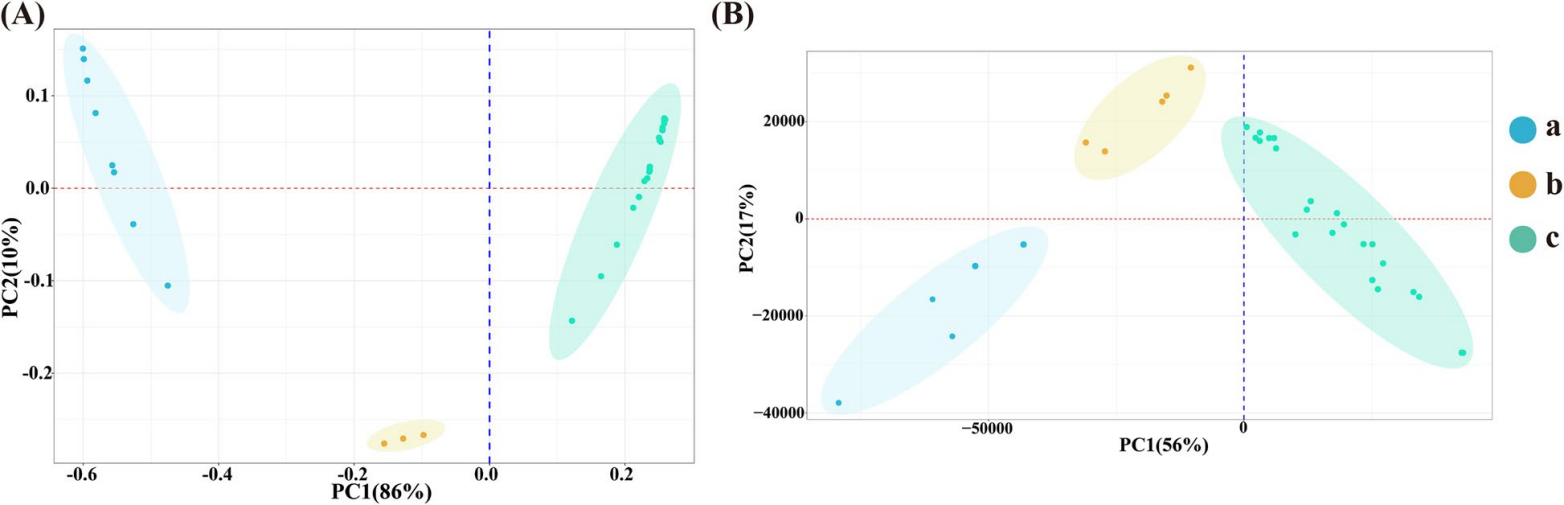
PCA score plot in monosaccharide content and transcriptome profiles of ginseng samples. **A** PCA score plot in nine monosaccharides content of four ginseng cultivars. Each point in PCA score plot representing an independent biological replicate. a, the roots sample from GL, CM and BT; b, the roots sample from SZ; c, the stems and leaves from GL, CM, BT, and SZ. **B** PCA score plot in transcriptome profile of four ginseng cultivars. a, the roots sample from GL and CM; b, the roots sample from BT and SZ; c the stems and leaves from GL, CM, BT, and SZ.

This study determined the content of nine monosaccharides in the roots, stems, and leaves of four cultivars by High Performance Liquid Chromatography (HPLC). The total content of nine monosaccharides in roots was higher than that in stems and leaves, and that in stems and leaves were similar (Fig. 1). Among those samples, the total content of nine monosaccharides was varied. In the roots, the total content of nine monosaccharides was the highest in CM, followed by the BT and GL, and the lowest in SZ (*P* < 0.05, Fig. 1 A). The total content of nine monosaccharides was similar to the four cultivars in the stems and leaves (*P* < 0.05, Fig. 1B, C). In the roots, only Glc was significantly different among the four cultivars, the most in CM and the least in BT (*P* < 0.05, Additional file 3: Table S2). In the stems, only GalA was the most abundant in GL (*P* < 0.05), while similar in the other three cultivars (Additional file 3: Table S2). No leaves were significantly different (Additional file 3: Table S2). PCA score plot of four cultivars based on the nine monosaccharides content, and the results showed that the first two principal components (PC) explained 96% of the total variation (PC1 = 86%, PC2 = 10%). All stems and leaves from four cultivars were clustered together, and clearly separated from roots, indicating significant differences in polysaccharides accumulation among tissues (Fig. 2 A). In addition, Glc was the most component of monosaccharides in all samples (*P* < 0.05, Table 1).

**Table 1:** The content of nine monosaccharides in each sample

		GL (mg/g)			CM (mg/g)			SZ (mg/g)			BT (mg/g)	
	Root	Stem	Leaf	Root	Stem	Leaf	Root	Stem	Leaf	Root	Stem	Leaf
Man	0.0290b	0.0277b	0.0339b	0.0358b	0.0267b	0.0277b	0.0268b	0.0271b	0.0272b	0.0307b	0.0276b	0.0288b
GlcA	0.0587b	0.0508b	0.0501b	0.0657b	0.0500b	0.0498b	0.0588b	0.0502b	0.0503b	0.0681b	0.0510b	0.0524b
Rha	0.0382b	0.0292b	0.0292b	0.0477b	0.0283b	0.0279b	0.0340b	0.0281b	0.0287b	0.0455b	0.0288b	0.0306b
GalA	0.0389b	0.0304b	0.0298b	0.0497b	0.0293b	0.0324b	0.0365b	0.0294b	0.0323b	0.0488b	0.0294b	0.0304b
Glc	15.6215a	1.5288a	1.3461a	17.2789a	1.1827a	1.5607a	4.2758a	1.5825a	1.1975a	15.0773a	1.3242a	1.4523a
Gal	0.0513b	0.0631b	0.0639b	0.0561b	0.0537b	0.0895b	0.0481b	0.0539b	0.0722b	0.0499b	0.0578b	0.0868b
Xyl	0.0394b	0.0349b	0.0337b	0.0388b	0.0334b	0.0334b	0.0361b	0.0346b	0.0335b	0.0373b	0.0346b	0.0346b
Ara	0.0452b	0.0470b	0.0485b	0.0475b	0.0443b	0.0557b	0.0441b	0.0450b	0.0511b	0.0458b	0.0465b	0.0591b
Fuc	0.0451b	0.0448b	0.0450b	0.0451b	0.0448b	0.0452b	0.0449b	0.0449b	0.0451b	0.0452b	0.0450b	0.0451b

Different lowercase letters indicate significant differences of roots, stems, and leaves for four cultivars among the nine monosaccharides at the 0.05 level

### Differential expression genes in four cultivars

To comprehensively investigate the differences in gene expression levels of four ginseng cultivars, we performed transcriptome sequencing for GL, CM, SZ, and BT. We sequenced 33 libraries from four cultivar samples’ roots, stems, and leaves (Additional file 4: Table S3). For further analysis, low-quality sequences were filtered out, and 241.37 G clean reads were obtained from the 33 libraries. Using the ‘Chunpoong’ genome as a reference genome, we mapped an average of 79.27%, 78.33%, and 77.65% of clean reads for the roots, stems, and leaves, respectively (Additional file 4: Table S3). The heatmaps of PCC values showed that the biological replicates had similar expression patterns and an extremely high PCC value (PCC > 0.80), except for BT1_R (average PCC = 0.44; Additional file 1: Fig. S1). Therefore, sample BT1_R was discarded from all subsequent analyses.

Based on the transcriptomic profile, PC1 and PC2 together explained 56% and 17% of gene expression variances among all samples, respectively. It is worth noting that the PCA score map showed stems and leaves tissue were clustered together, significant segregation from roots, indicating that gene expression at the transcriptome level responded to tissue changes, which was consistent with the results of the polysaccharides content (Fig. 2B). Differentially expressed genes (DEGs) were identified from the roots, stems, and leaves in each ginseng cultivar. We found that the number of DEG was the highest in roots (3, 638) and stems (2, 467) between GL and SZ comparison group, while the number of DEGs was the highest in leaves (2, 938) between CM and BT comparison groups. The fewest DEGs were detected between BT and SZ comparison groups in roots (429) and leaves (1,435). In stems, there were the fewest DEGs between GL and BT comparison groups (941) (Additional file 1: Fig. S2). The number of DEGs was 4,10, and 4 in the comparison groups from a different root, stem, and leaf cultivars, respectively (Additional file 1: Fig. S2).

**Fig. 3:**
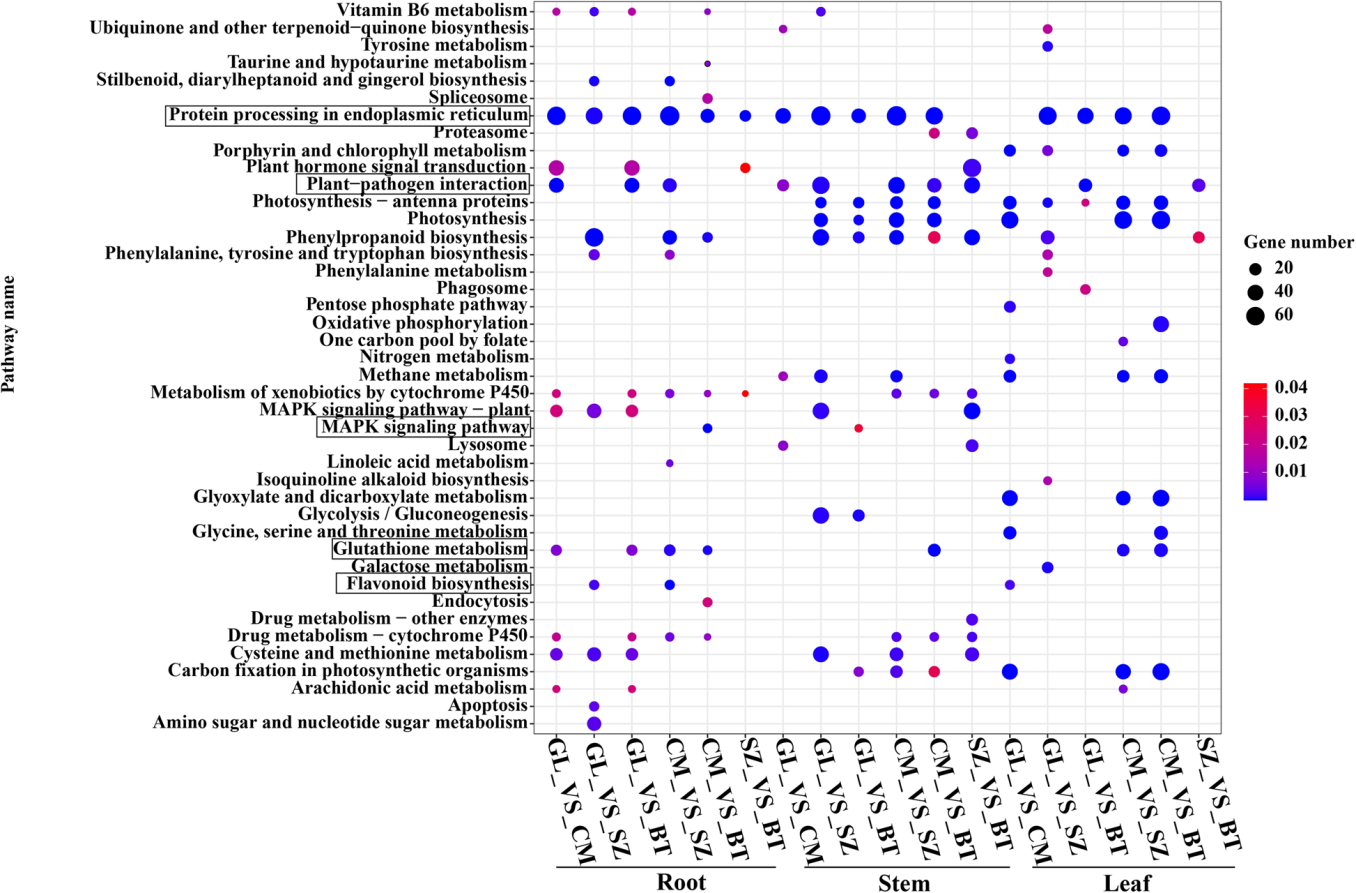
Kyoto Encyclopedia of Genes and Genomes (KEGG) pathway enrichment of differentially expressed genes (DEGs) in each of the comparison groups

Next, to better understand the functions of DEGs, we performed the Kyoto Encyclopedia of Genes and Genomes (KEGG) enrichment analysis and gene ontology (GO) category enrichment analysis. The DEGs of roots, stems, and leaves were enriched in some secondary metabolic pathways, such as glutathione metabolism, flavonoid biosynthesis, MAPK signaling pathway, protein processing in endoplasmic reticulum, and plant-pathogen interaction et al. (Fig. 3). In the GO enrichment analysis, the enriched terms of the DEGs included response to chitin, response to high light intensity, photosynthesis, and phenylalanine ammonia-lyase activity in roots, stems, and leaves comparison groups (Additional file 1: Figs. S3, S4, S5).

### Ginseng polysaccharides biosynthetic pathway

**Fig. 4:**
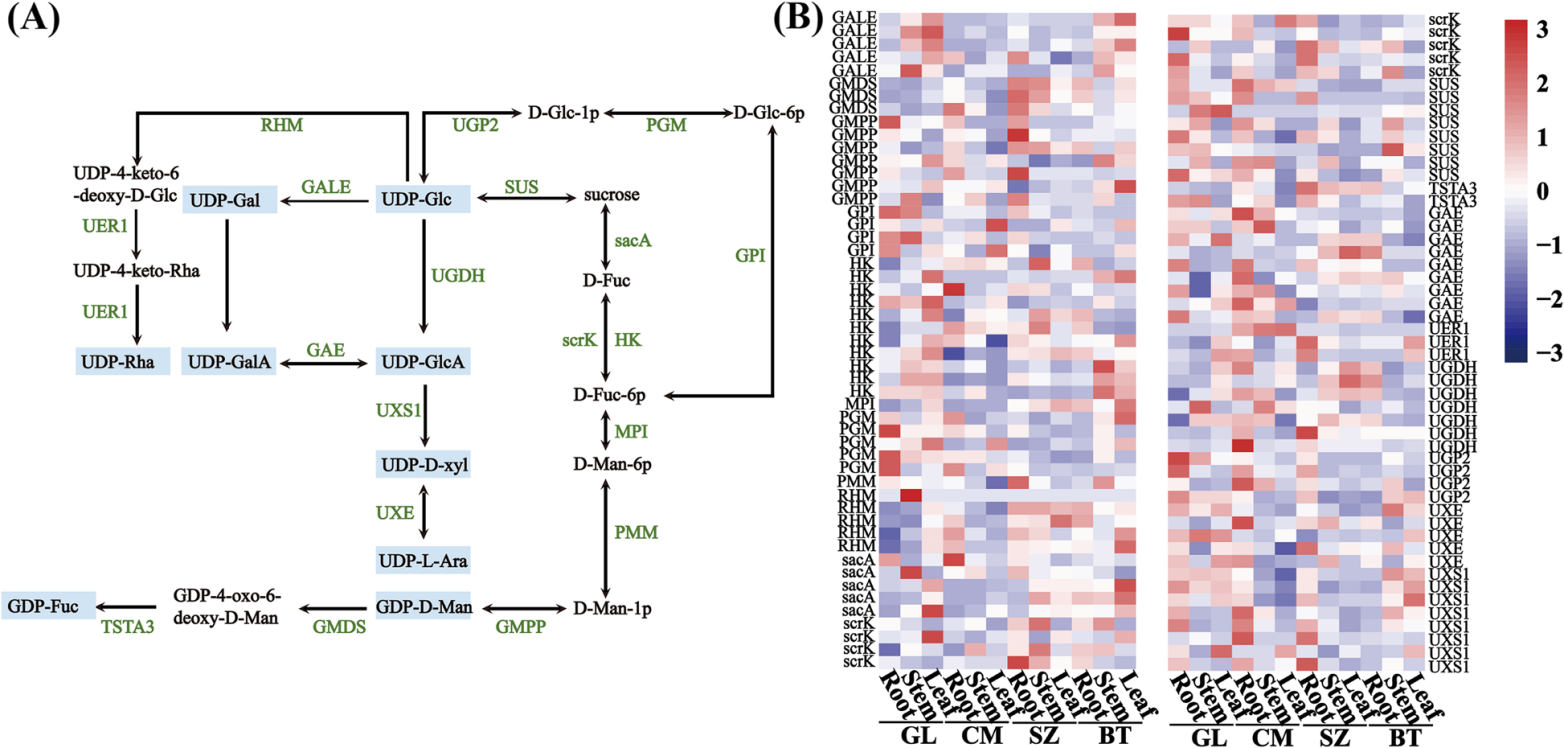
Putative pathway for polysaccharides biosynthesis and the expression of related gene in ginseng. **A** Polysaccharides biosynthetic pathway. The green word represented enzyme involved in polysaccharides, and blue frames represented polysaccharides products. **B** Expression level of genes in polysaccharides biosynthesis pathway in different cultivars and different tissues. The red and blue blocks represent high and low expression levels, respectively. D-fructose-6p, D-fructose-6phosphate; D-man-6p, D-mannan-6phosphate; D-man-1p, D-mannan-1phosphate; GDP-D-Man, GDP-D-mannose; GDP-4-oxo-6-deoxy-D-Man, GDP-4-oxo-6-deoxy-D-mannose; GDP- Fuc, GDP-L-fuc; D-glc-1p, D-glucose-1phosphate; D-glc-6p, D-glucose-6phosphate; UDP-Glc: UDP-glucose; UDP-Gal, UDP-galactose; UDP-glcA, UDP-glucuronate; UDP-D-xyl, UDP-D-xylose; UDP-L-Ara, UDP-L-arabinose; UDP-GalA, UDP-D-galacturonate; UDP–4-keto-6-deoxy-D-Glc, UDP–4-keto-6-deoxy-D-glucose; UDP-4-keto-Rha, UDP-4-keto-rhamnose; UDP-Rha, UDP-rhamnose; GAE, UDP-glucuronate 4-epimerase; GALE, UDP-glucose 4-epimerase; GMDS, GDP-mannose 4,6-dehydratase; GMPP, mannose-1-phosphate guanylyltransferase; GPI, glucose-6-phosphate isomerase; HK, hexokinase; MPI, mannose-6-phosphate isomerase; PGM, phosphoglucomutase; PMM, phosphomannomutase; RHM, UDP-glucose 4,6-dehydratase; sacA, beta-fructofuranosidase; scrK, fructokinase; SUS, sucrose synthase; TSTA3, GDP-L-fucose synthase; UXE, UDP-arabinose 4-epimerase; UGDH, UDP-glucose 6-dehydrogenase; UGP2, UTP–glucose-1-phosphate uridylyltransferase; UXS1, UDP-glucuronate decarboxylase

To identify genes involved in the biosynthetic pathway of ginseng polysaccharides, we annotated the genes related to starch and sucrose metabolism (ko00500), fructose and mannose metabolism (ko00051), galactose metabolism (ko00052), and amino sugar and nucleotide sugar metabolism (ko00520). Based on the main monosaccharide components in ginseng polysaccharides, we outlined potential biosynthetic pathways for forming ginseng polysaccharides from sucrose. Sucrose was converted to D-fructose, then D-fructose-6phosphate (D-fructose-6p) to D-mannan-6p indirectly, and from D-mannan-1p to GDP-D-Man; subsequently, GDP-4-oxo-6-deoxy-D-Man to GDP-L-Fuc. In addition, sucrose was instantaneously transformed into UDP-Glc, UDP-glcA to UDP-D-xyl, and then UDP-D-xyl into UDP-L-Ara. Moreover, UDP-Glc was converted to D-glucose-6p and then to GDP-Fuc. In addition, UDP-Gal was also directly derived from UDP-Glc, and UDP-4-keto-6-deoxy-D-glc was converted to UDP-4-keto-Rha and UDP-Rha (Fig. 4 A). The components of ginseng polysaccharides included Glc, Gal, Rha, mannose (Man), Xyl, Ara, Glc-acid (GlcA), Gal-acid (GalA), and fucose (Fuc) (Fig. 4 A).

### Relationship between gene expression and metabolite accumulation in polysaccharides biosynthesis

In the biosynthetic pathway of ginseng polysaccharides, we found 102 genes encoding 19 key enzymes that control the synthesis of ginseng polysaccharides. According to structural genes extracted from polysaccharides biosynthesis pathway, the expression levels of these genes in different samples were significantly districting. We found that most of the genes encoded UTP-glucose-1-phosphate uridylyltransferase (UGP2), phosphoglucomutase (PGM), and sucrose synthase (SUS) in the root of GL and CM expressed at higher levels than that of BT and SZ. The expression level of genes encoded UDP-glucose 4-epimerase (GALE) were higher in stems and leaves of GL and SZ than of CM and BT. In addition, the genes encoded mannose-6-phosphate isomerase (MPI), and GDP-mannose 4,6-dehydratase (GMDS) were expressed at the highest levels in the stems of SZ. Other genes in the pathway synthesize polysaccharides, such as UDP-glucuronate decarboxylase (UXS1), UDP-arabinose 4-epimerase (UXE), UDP-glucose 6-dehydrogenase (UGDH), and hexokinase (HK), the expression levels varied in different tissues across samples (Fig. 4B). These results suggested that the synthesis of ginseng polysaccharides may be a pathway for multigene cooperative regulation.

The correlation analysis of polysaccharides synthesis related genes and content of polysaccharides suggested that 17 enzymes [PGM, fructokinase (scrK), beta-fructofuranosidase (sacA), UXE, UXS1, mannose-1-phosphate guanylyltransferase (GMPP), UGP2, GALE, MPI, GDP-L-fucose synthase (TSTA3), SUS, UDP-glucuronate 4-epimerase (GAE), HK, phosphomannomutase (PMM), UGDH, GMDS, and glucose-6-phosphate isomerase (GPI)] were correlated to the content of monosaccharide and total polysaccharides. In addition, the expression of genes encoding scrK (Pg_S0635.5, Pg_S1306.14, Pg_S1495.1, Pg_S0588.13, Pg_S5155.1, Pg_S2241.31, and Pg_S3153.2) was positively corrected to most of the monosaccharide content and total polysaccharides content, which of HK (Pg_S4434.4, Pg_S3346.1, Pg_S4929.12, and Pg_S0234.21) was negatively correlated (Additional file 5: Table S4).

### Co-expression modules related to the content of polysaccharides

**Fig. 5:**
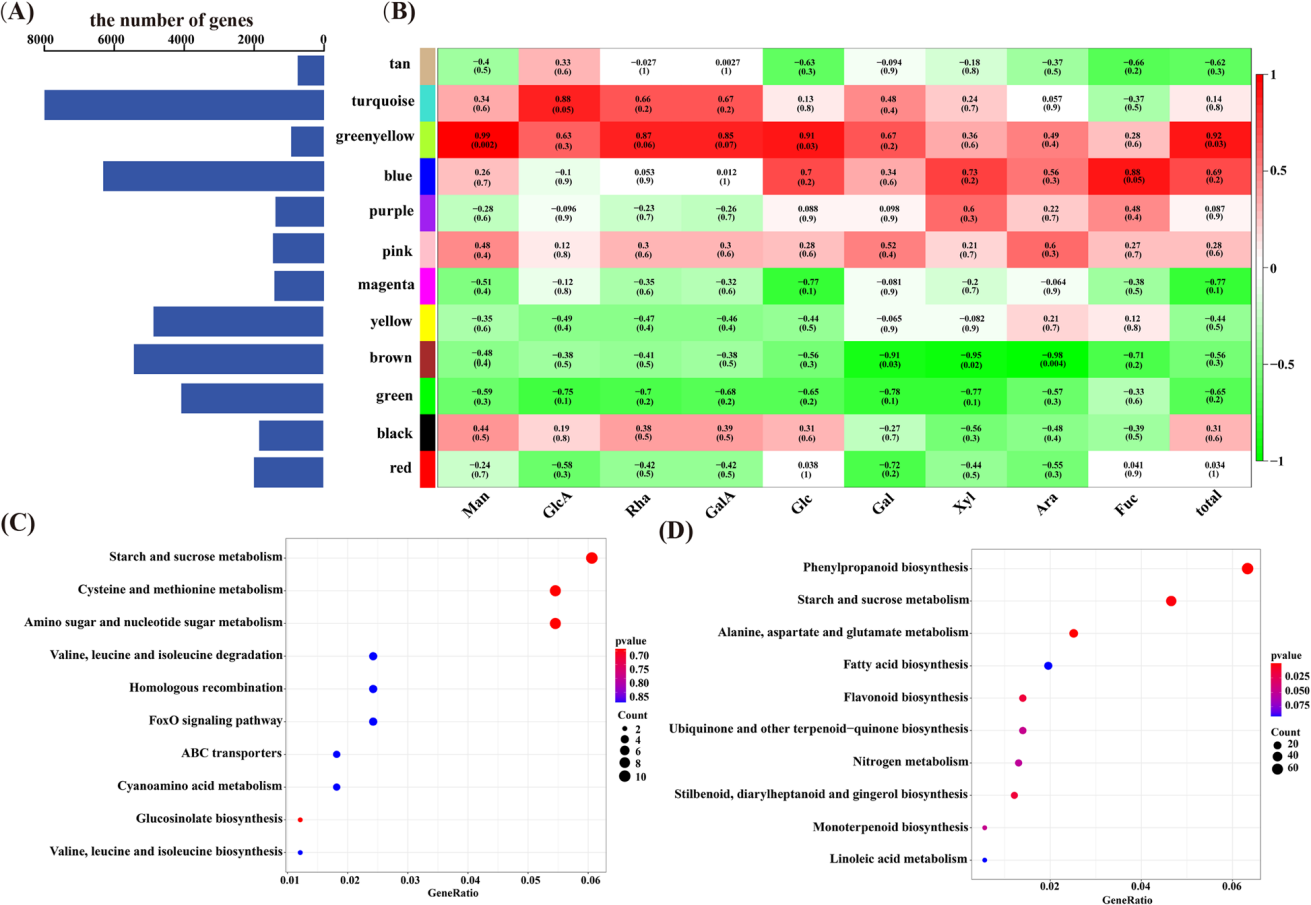
Results of the gene co-expression network analysis based on WGCNA. **A** The number of genes contained in each module. **B** Correlation coefficient between polysaccharides and module eigengenes presented with a color scale with red and green representing positive and negative correlations, respectively. Glc, Gal, Rha, Man, Xyl, Ara, GlcA, GalA, Fuc and total represent glucose, galactose, rhamnose, mannose, xylose, arabinose, glucuronic acid, galacturonic acid, fucose and total polysaccharides, respectively. The different colors of the y-axis represent different modules. **C** and **D** represent KEGG annotation analysis of genes in the greenyellow and brown modules. The x-axis represents the gene ratio (annotation number/background number), and the y-axis represents the pathway name

In our study, the difference in the total content of nine monosaccharides between CM and SZ was most obvious in the root, and the number of DEGs were the most of the root in CM_vs_SZ. We screened a co-expression module by Weighted Gene Co-Expression Network Analysis (WGCNA) of 46, 807 genes and the content of nine monosaccharides, which come from the root of CM and SZ. This analysis identified 12 co-expression modules containing 919 to 9, 747 genes (Fig. 5 A). Pearson correlation analysis between module eigengenes and the Glc, Gal, Rha, Man, Xyl, Ara, GlcA, GalA, Fuc, and total content of nine monosaccharides (total), indicated the biological importance of the two modules (greenyellow and brown). The greenyellow module obviously correlated with the content of Man (*r* = 0.99, *P* = 0.002), Glc (*r* = 0.91, *P* = 0.03) and total (*r* = 0.92, *P* = 0.03). The brown module was highly correlated with the content of Gal (*r* = -0.91, *P* = 0.03), Xyl (*r* = -0.95, *P* = 0.02), and Ara (*r* = -0.98, *P* = 0.004) (Fig. 5B). These results suggested that the two modules obviously correlated with the content of polysaccharides accumulation in ginseng.

The KEGG annotation showed that genes in these two modules were mainly related to metabolite pathways, such as phenylpropanoid biosynthesis (ko00940), starch and sucrose metabolism (ko00500), and amino sugar and nucleotide sugar metabolism (ko00520) (Fig. 5 C, D). In addition, a large number of genes for polysaccharides biosynthesis were found in these two modules, such as genes encoding PGM, GPI, scrK, UGP2, GMPP, PMM, GALE, sacA, and SUS (Table 2).

**Table 2:** Genes are involved in the ginseng polysaccharide synthesis pathway in the module

Module	Gene_id	Gene family
greenyellow	Pg_S2017.3	PGM
	Pg_S7036.4	GPI
	Pg_S1306.14	scrK
	Pg_S1124.2	UGP2
	Pg_S0167.13	GPI
brown	Pg_S0635.5	scrK
	Pg_S0953.13	GMPP
	Pg_S4516.21	PMM
	Pg_S0889.28	GALE
	Pg_S2031.4	sacA
	Pg_S0897.14	GALE
	Pg_S1886.12	GMPP
	Pg_S0061.8	sacA
	Pg_S0219.46	UGDH
	Pg_S3876.17	sacA
	Pg_S3604.8	GALE
	Pg_S3338.6	GMPP
	Pg_S2762.11	SUS
	Pg_S0455.9	GMPP

*PGM* Phosphoglucomutase, *GPI* Glucose-6-phosphate isomerase, *scrK* Fructokinase, *UGP2* UTP–glucose-1-phosphate uridylyltransferase, *GMPP* Mannose-1-phosphate guanylyltransferase, *PMM* Phosphomannomutase, *GALE* UDP-glucose 4-epimerase; *sacA* Beta-fructofuranosidase, *SUS* Sucrose synthase

**Fig. 6:**
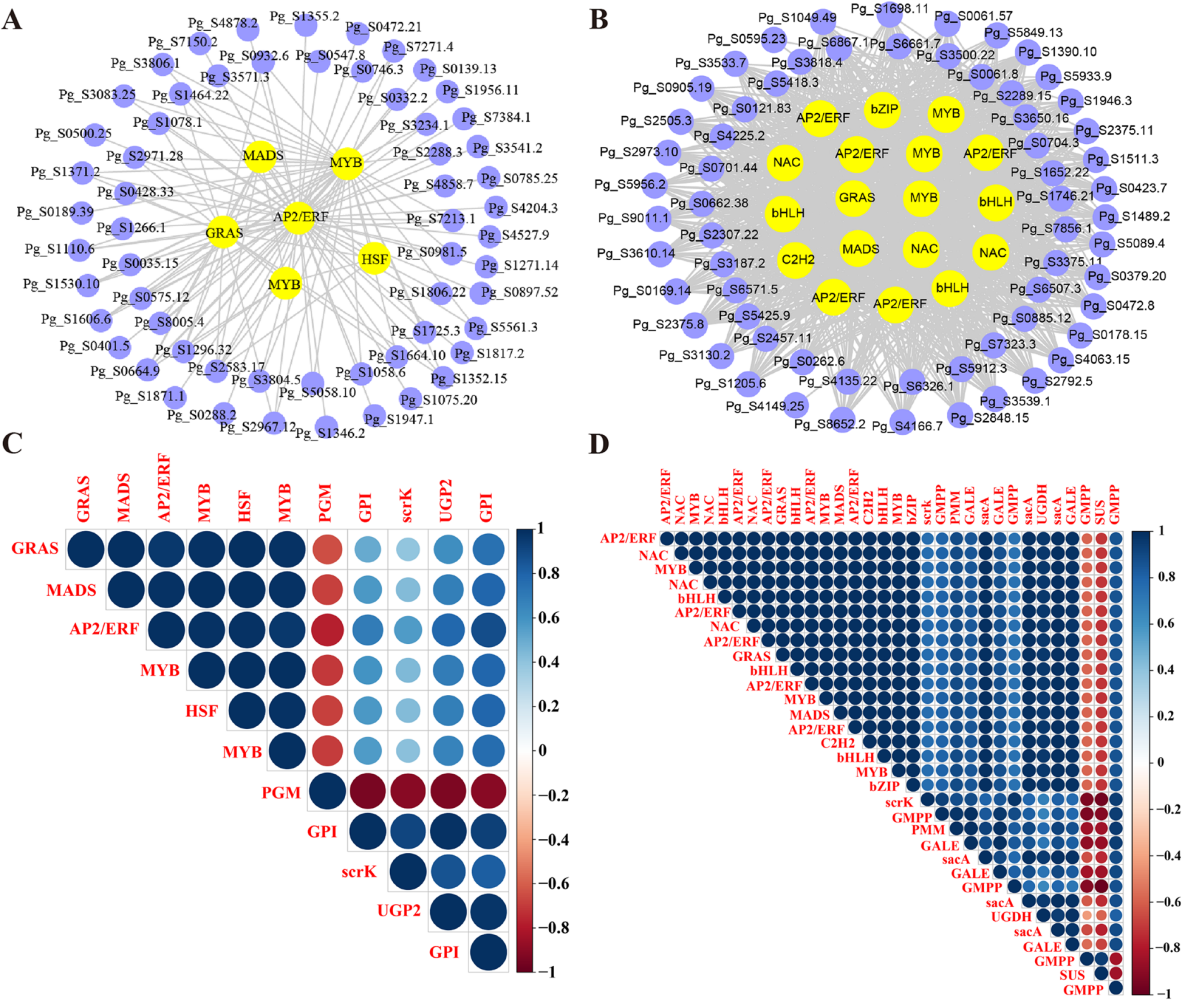
Construction of gene regulation network and correlation analysis. **A** Gene co-expression subnetwork of the greenyellow module. **B** Gene co-expression subnetwork of the brown module. **C**, Correlation analysis of TFs and polysaccharides synthesis genes in the greenyellow module. **D** Correlation analysis of TFs and polysaccharides synthesis genes in the brown module. Network was reconstructed by edge weight cutoff = 0.50 and visualized by Cytoscape. The red and blue blocks represent positive and negative correlations, respectively

In order to find the key regulatory TFs related to polysaccharides biosynthesis from these two modules, we constructed a gene correlation network for each module by Cytoscape. In the greenyellow module, 6 TFs were identified, GRAS (Pg_S2354.13), MADS (Pg_S4852.3), AP2/ERF (Pg_S4672.9), MYB (Pg_S1414.8 and Pg_S4889.3), and HSF (Pg_S3558.9) (Fig. 6 A). It was found that the expression level of these TFs was highly related to that of GPI, PGM, and UGP2 (Fig. 6 C). A total of 18 genes encoding 8 TFs were found in the brown module, including MYB (Pg_S3722.2, Pg_S7293.3, and Pg_S2010.18), bZIP (Pg_S1242.23), AP2/ERF (Pg_S6406.9, Pg_S3071.2, Pg_S0253.9, Pg_S3048.23, and Pg_S4277.1), bHLH (Pg_S0724.61, Pg_S0734.14, and Pg_S0817.8), NAC (Pg_S2569.3, Pg_S1059.27, and Pg_S3248.6), MADS (Pg_S1390.1), GRAS (Pg_S0325.10), and C2H2 (Pg_S6161.2) (Fig. 6B). The expression of these TFs was highly related to that of all the genes that encode scrK, GMPP, PMM, GALE, sacA, and SUS in the brown module, except for Pg_S3338.6 encoding GMPP (Fig. 6D). These results suggested that these TFs might regulate the expression of genes related to ginseng polysaccharides synthesis.

### qRT-PCR validation

To verify the gene expression levels produced by RNA-Seq, we performed qRT-PCR analysis on ten independent samples. We selected 10 DEGs in six compared groups, and as expected, 10 DEGs exhibited similar expression tendencies. Finally, the results show that the RNA-Seq data are accurate and useful (Additional file 1: Fig. S6).

## Discussion

### Effects of cultivars on polysaccharides content

Ginseng polysaccharides are the active ingredient in ginseng, which have anti-tumor, anti-inflammation, anti-oxidation and immunomodulatory effects [24–26]. Our study found that the total content of nine monosaccharides was significantly higher in roots than in stems and leaves. Previous studies showed that polysaccharides were produced in different tissues of ginseng, such as roots, stems, leaves, flowers, and fruits, but polysaccharides were highest in roots [25, 27]. The result of PCA also showed that all stems and leaves from four cultivars were clustered together, but were obviously separated from the roots, suggesting accumulation of ginseng polysaccharides was different in various tissues. Moreover, the content of ginseng polysaccharides was less in the stem and leaf of four cultivars. Here it is speculated that the main synthetic tissue of ginseng polysaccharides in the roots, or ginseng polysaccharides are synthesized in different tissues of ginseng and eventually transported to the roots, just like ginsenosides [28]. It is necessary to collect different tissues of ginseng at different growth stages to determine and analyze polysaccharides to test this hypothesis in the future.

Under environmental stress and nutrient restriction, plants accumulate secondary metabolites to improve their defense response [29]. As a secondary metabolite, sugar metabolism is affected by various abiotic stresses, and plants regulate growth and development by regulating the accumulation of sugars in response to abiotic stresses, including cold stress [30, 31]. The total monosaccharides content of the two cultivars (‘Hayward’ and ‘Haegeum’) of kiwi fruit was increased by 4–5 times after the fruit was stored at 0℃ for 4 months [32].The total content of nine monosaccharides in the root of CM was the highest in this study. The high polysaccharides content of COMMON ginseng might be related to its high cold resistance [19]. Meanwhile, we found that the DEGs among different cultivars were mainly enriched in MAPK signaling pathway and plant-pathogen interaction pathways. Both metabolic pathways are involved in plant defense and immune systems to improve stress resistance [33, 34]. These data seem to support that the resistance of different ginseng cultivars also varied. In addition, Glc was significantly higher in CM than in the other three cultivars. Increasing evidence indicating that soluble sugars, including Glc and Fuc et al., were not only a source of energy, but also an osmoprotectant in plants, increasing water retention capacity by reducing the water potential of cells and thus improving plant cold resistance [30, 35]. Perhaps due to this mechanism, COMMON ginseng seems to be better adapted to cold stress than the other three cultivars.

### The key genes in the synthetic pathway of ginseng polysaccharides

The structural characteristics of ginseng polysaccharides have always attracted people’s interest [36, 37]. Many studies on the analysis of ginseng polysaccharides composition indicated that ginseng polysaccharides are composed of Glc, Gla, Ara, and Man etc. [13]. However, the biosynthetic pathway of ginseng polysaccharides has not been elucidated. Through the analysis of bioinformatics and monosaccharides content determination, we identified the key enzymes of ginseng polysaccharides biosynthesis. The results showed that the precursor of polysaccharides biosynthesis was sucrose. The synthetic pathway of ginseng polysaccharides is similar to that of *P. sibiricum*, *Poria cocos*, and *Hericium frutescens* [38–40]. Previous studies showed that despite the striking differences in the structure of polysaccharides, the biosynthesis process of polysaccharides was conserved [40, 41]. In the synthesis pathway of ginseng polysaccharides, 19 key enzymes were identified. The expression levels of 17 enzymes were significantly correlated with the content of polysaccharides, suggesting these enzymes are the main enzymes in synthesizing ginseng polysaccharides.

Most of the genes encoded in UGP2, PGM, and SUS were expressed at higher levels in the root of GL and CM than that of BT and SZ. Moreover, the polysaccharides content was significantly higher in GL and SZ roots than that in other tissues. During the growth of transgenic *Arabidopsis thaliana*, overexpression of UGP gene promoted the increase of sugar content in *A*. *thaliana* [42]. Xu et al. (2015) suggested the content of intracellular polysaccharide (IPS) and extracellular polysaccharide (EPS) in *Ganoderma lucidum* overexpressing the PGM gene were higher than those of the wild-type strain [43]. We concluded that UGP2, PGM, and SUS were key enzymes of Glc synthesis in the biosynthetic pathway of ginseng polysaccharides.

ScrK is a key enzyme that acts upstream in the biosynthesis of ginseng polysaccharides. It is responsible for converting D-fructose into D-fructose 6phosphate, and the activity of scrK effectively controls the accumulation of starch in tomato fruit [44]. HK is also the key enzyme involved in converting D-fructose to D-fructose-6p, and HK plays an indispensable role in sugar accumulation in apple fruits [45, 46]. Scrk and HK played a role in the same branches of the ginseng polysaccharides biosynthesis pathway, but they showed the opposite correlation pattern with polysaccharides content in this study. They might play a contrary role in the biosynthesis pathway of polysaccharides. In the polysaccharides synthesis pathway of *P. sibiricum*, HK and scrK also showed converse effects [38].

### Transcription factors acting on the regulation of polysaccharides content

Several external and internal factors influence gene expression through TFs that influence them and are bind to specific regions of the target gene promoter [47, 48]. In recent years, WGCNA analysis has been frequently used to identify specific genes and TFs to synthesis certain metabolites in plants [49, 50]. We identified two modules related to polysaccharides accumulation using WGCNA, including greenyellow and brown modules. Multiple polysaccharides biosynthesis-related genes and TFs exhibited specific interaction patterns to modulate the ginseng polysaccharides biosynthesis in these two modules.

In the greenyellow module, the expression of MYB (Pg_S1414.8 and Pg_S4889.3) and AP2/ERF (Pg_S4672.9) were highly related to genes for polysaccharides synthesis (PGM, GPI, and UGP2). The MYB transcription family is one of the largest transcription families, which have been reported to play various roles in the secondary metabolism of plants [51, 52]. In *Arabidopsis*, Chen et al. (2017) found that MYBS1 and MYBS2 regulated the expression of glucose-responsive genes in seeds [53]. The AP2/ERF transcription factors have two conserved amino acid sequences. It has been reported that AP2/ERF regulated the starch content by binding to rice starch regulator1 (RSR1), a TF of the rice protein family [54]. The greenyellow module obviously correlated with the content of Glc. Meanwhile, PGM, GPI, and UGP2 are the main branches of Glc synthesis in the pathway of ginseng polysaccharides. Thereby, MYB and AP2/ERF might be the key TFs regulating the expression of genes that synthesize Glc (PGM, GPI, and UGP2), leading to higher the content of Glc.

In the brown module, we also found the other two TFs expected MYB and AP2/ERF, including bZIP (Pg_S1242.23) and NAC (Pg_S2569.3, Pg_S1059.27, and Pg_S3248.6), which were highly related to all the genes for polysaccharides synthesis (scrK, GMPP, PMM, GALE, sacA, and SUS). The bZIP identified in maize could affect the physiological and biochemical characteristics of maize endosperm, and its overexpression changed the expression of starch biosynthesis genes in the endosperm [55]. The NAC family had many members and diverse functions and had been confirmed to play the core role in starch biosynthesis in plants [56–58]. A novel transcription factor TaNAC019-A1, could directly bind to the ‘ACGCAG’ motif in the promoter regions of ADP-glucose pyrophosphorylase small subunit 1 region and inhibit its expression, thereby influencing starch synthesis in the wheat endosperm [59]. These TFs perhaps control the synthesis of ginseng polysaccharides by regulating the genes of ginseng polysaccharides synthesis.

## Conclusion

Combining transcriptome and polysaccharides content analysis of four ginseng cultivars explores the ginseng polysaccharides synthesis pathway and regulatory network. HPLC determined the content of nine monosaccharides, and the result showed that the total contents of nine monosaccharides were highest in the roots. Moreover, the content of nine monosaccharides in the roots of the four ginseng cultivars was different, but similar in the stems and leaves. Among monosaccharides, Glc is the most component of monosaccharides for all samples. In total, we identified 19 potential enzymes for the synthesis of ginseng polysaccharides, of which 17 enzymes were significantly associated with polysaccharides content. By WGCNA analysis, the expression of PGM, GPI, UGP2, scrK, GMPP, PMM, GALE, sacA, and SUS were correlations with that of MYB, AP2/ERF, bZIP, and NAC TFs. These TFs might regulate the genes of ginseng polysaccharides synthesis. These results revealed the biosynthetic mechanism of ginseng polysaccharides and provides a scientific basis for the research of ginseng polysaccharides.

## Methods

### Plant materials

Six-year-old roots, stems, and leaves from four *P. ginseng* cultivars [GAOLI ginseng (GL), COMMON ginseng (CM), SHIZHU ginseng (SZ), and BIANTIAO ginseng (BT)] were used in this study (Additional file 2: Table S1). Three biological replicates were collected for each ginseng cultivar (CM had only two replicates) (Additional file 2: Table S1). Ginseng root, stem, and leaf material were harvested in summer when the fruit was ripe. The root, stem, and leaf tissues were collected from the top, middle, and bottom of each sampled ginseng root, stem, and leaf, respectively, and preserved in liquid nitrogen for transcriptomics analyses. The remaining root, stem, and leaf on each sample were collected, dried at 55℃ and used for polysaccharides extraction.

### Extraction and determination of polysaccharides

Dry root, stem, and leaf powder of ginseng (50 mg) were used to extract ginseng polysaccharides, according to the method of Zhao et al. [60]. The extracted polysaccharides powder was transferred to a 10 ml top screw cap bottle, 4 mL of trifluoroacetic acid (TFA) was added to the bottle (2 mol L^− 1^), then the bottle mouth was closed, followed by hydrolysis at 110 °C for 3 h. After cooling to room temperature, methanol was added to the bottle to remove excess TFA, and the process was repeated five times, whereafter, 500 µL NaOH (0.3 mol L^− 1^) and 500 µL methanol − 1-phenyl-3-methyl-5-pyrazolone (PMP) (0.5 mol L^− 1^) were added to the bottles, and reacted in a 70 °C water bath for 40 min. After static cooling, added 500 µL HCL (0.3 mol L^− 1^). Finally, an equal volume of chloroform was added for extraction, shaken and stand still to remove the organic phase, and repeated the operation three times. The supernatant was filtered through 0.22 μm membrane and analyzed by HPLC (Agilent Technologies, USA). Wang et al. (2022) determined by gas chromatography-mass spectrometry (GC-MS) ginseng polysaccharide were composed of Ara, Rha, Fuc, Xyl, Man, Gal, and Glc [61]. In addition, the polysaccharide composition of many species was found to include GlcA acid and GalA [62–64]. Therefore, these nine monosaccharides were selected for the following analysis. Standard monosaccharides Glc, Gal, Rha, Man, Xyl, Ara, GlcA, GalA, and Fuc (Solarbio, China) were analyzed by the same experimental steps. The mobile phase was selected as 0.05 M phosphate (A) in water, and acetonitrile (B), and the gradient of 8% B for 45 min were used with a flow rate of 1.0 mL min^− 1^. The detection wavelength was set to 250 nm. The target chromatographic peaks were identified by comparing the retention time with their standards. Quantification was calculated by peak integration using the external standard method. Each sample was repeated three times. To realize the relationship of polysaccharides content among samples, principal coordinate analysis (PCoA) was implemented following R package models (vegan and ape).

### RNA extraction and sequencing

Total RNA was isolated from ginseng roots, stems, and leaves using the TRIzol Reagent (Invitrogen, USA) following the manufacturer’s instructions, and the quantity of the RNA was assessed by NanoDrop 2000 (Thermo Scientific, USA) and gel electrophoresis. The mRNA was purified from total RNA using poly-T oligo-attached magnetic beads. After quality control (Agilent 2100 Bioanalyzer; ABI StepOnePlus Real-Time PCR System (TaqMan Probe), USA) and library preparation, cDNA was sequenced in paired-end mode, 150 bp length (PE150) using Illumina HighSeq Xten.

### RNA-seq data analysis

The raw reads were filtered by removing adaptor sequences and low-quality sequences (Phred quality scores < 10) using Trimmomatic (v0.36) [65]. The high-quality reads were then mapped to the reference genome of *P. ginseng* “Chunpoong” [66] (http://ginsengdb.snu.ac.kr) with HISAT2 software (v2.1.0) [67]. The uniquely mapped reads were retained and used for further analysis. The gene function was annotated to GO databases and KEGG databases using EggNOG (v5.0) and KAAS (v2.1), respectively [68, 69]. Transcription factors (TFs) were annotated and classified using iTAK online (v1.6) based on the Plant Transcription Factor Database (PlantTFDB v5.0) [70].

### Identification of DEGs

Gene expression levels were estimated using RSEM (v1.3.0), and the TPM (transcript per million) value was used to quantify gene expression levels [71]. Pearson correlation coefficients (PCC) of expression levels were calculated between each pair of ginseng cultivars using R package (v4.2.0), and the PCC values < 0.5 across samples were removed. DEGs were identified using DESeq2 (v1.28.1) in R software (v4.2.0) (|log2FoldChange| > 1 and *P* < 0.05) [72]. When log2FoldChange > 1, DEG was up-regulated. In contrast, for log2FoldChange < -1, it was considered a downward adjustment. Based on gene expression, principal component analysis (PCA) was performed with R packages (prcomp), which was used to reveal the relationship among samples. To identify significantly enriched GO terms and KEGG pathways of DEGs with the entire transcriptome background, GO and KEGG enrichment analyses were performed with *P* < 0.05 by clusterProfiler (v3.16.1) using R package (v4.2.0) [73].

### Analysis of candidate genes involved in ginseng polysaccharide biosynthesis

Based on the composition of ginseng polysaccharides, which were composed of nine monosaccharides, including Glc, Gal, Rha, Man, GlcA, GalA, and Fuc et al. [7, 12], we determined candidate genes involved in biosynthetic pathway of ginseng polysaccharides on the base of the KEGG pathway annotation, including starch and sucrose metabolism (ko00500), fructose and mannose metabolism (ko00051), galactose metabolism (ko00052), and amino sugar and nucleotide sugar metabolism (ko00520) [74]. All potential genes were classified by BLAST using the KEGG database (v2.7.1) [74].

### Statistical analysis

The content of nine monosaccharides were expressed as mean ± SD. One-way anOVA (ANOVA) was used to analyze differences in content of total polysaccharides between groups and the differences in the contents of nine monosaccharides within the same individual. *P* < 0.05 was considered statistically significant [75]. We examined the relationships between the genes involved in polysaccharides biosynthesis by correlation analysis and the variations of nine monosaccharides and total nine monosaccharides’ contents in all tissues of four ginseng cultivars. The correlation was performed using the R package, with significance levels as the *p*-value cutoff (*P* < 0.05).

### WGCNA and visualization of gene networks

In order to analyze the regulatory mechanism of ginseng polysaccharides biosynthesis and to explore possible transcriptional factors (TFs) involved, weighted gene co-expression network analysis (WGCNA) was performed. A total of 46, 807 genes were used to perform WGCNA analysis in R packages (v4.2.0) [76]. Next, WGCNA network construction and module detection were performed by the “blockwiseModules” function. Modules were identified using the power = 12, the minModuleSize = 30, and the mergeCutHeight = 0.25, and other parameters using default settings.

To identify modules associated with the content of ginseng polysaccharides, we calculated the module eigengene by the genes of each module and correlated these eigengenes with the ginseng cultivars and tissues. Modules with *P* < 0.05 and |correlation coefficients| (*r*) > 0.90 were considered as significant related modules. In order to further investigate the key genes in these modules, the genes in significant related module were analyzed based on KEGG pathway annotation. Cytoscape (v3.7.0) was used to visualize the most significantly correlated genes with a WGCNA edge weight > 0.50, then we considered the top 20% of the connected genes as hub genes in the module [77]. The correlation analysis between genes in the pathway of polysaccharides synthesis and hub genes in the module was analyzed using R package (v4.2.0).

### RT-qPCR validation

To verify the differentially expressed transcripts from RNA-Seq, we used quantitative real-time PCR assay. We selected 10 genes to validate the reliability of the transcriptome data. These gene-specific primers were designed for ten genes by Primer Premier 6.0, and synthesized by Sangon Biotech Co., Ltd. (Shanghai, China). In experiments for cultivars-specific expression confirmation, the relative expression levels of the candidate genes were calculated with the 2^−ΔΔCT^ method using GAPDH as the internal reference gene [78]. PCR amplification was performed: 95℃ for 5 min, 40 cycles of 95℃ for 30 s, and 60℃ for 30 s, and with a dissociation stage of 95℃ for 15 s, 60℃ for 60 s, and 95℃ for 15 s. All the reactions in all experiments were repeated three times (ThermoFisher Scientific).

## Supplementary Information

**Additional file 1: Fig. S1.** The heatmap of Pearson correlation coefficients (PCC) among biological repeats of each accession. Red represents the high correlation; blue represents the low correlation. R means root; S means stem; L means leaf. GL means GAOLI ginseng; CM means COMMON; SZ means SHIZHU ginseng; BT means BIANTIAO ginseng (BT). **Fig. S2.** The number of differentially expressed genes (DEGs) in each comparison group of different cultivars in the same tissue. GL means GAOLI ginseng; CM means COMMON; SZ means SHIZHU ginseng; BT means BIANTIAO ginseng (BT). **Fig. S3.** Gene Ontology (GO) enrichment analysis was performed for all DEGs from root in each comparison group. The bottom x-axis indicates represents the enrichment ratio of DEG (sample number/background number), and the y-axis represents each detailed classification of GO. **Fig. S4.** Gene Ontology (GO) enrichment analysis was performed for all DEGs from stem in each comparison group. The bottom x-axis indicates represents the enrichment ratio of DEG (sample number/background number), and the y-axis represents each detailed classification of GO. **Fig. S5.** Gene Ontology (GO) enrichment analysis was performed for all DEGs from leaf in each comparison group. The bottom x-axis indicates represents the enrichment ratio of DEG (sample number/background number), and the y-axis represents each detailed classification of GO. **Fig. S6.** Validation of the RNA-seq results by qRT-PCR, Bars show means of Log2 TPM (counts per length of transcript sequence per million mapped fragments) value and Log2 qRT-PCR value of two and three biological replicates, respectively. R: root; S: stem; L: leaf.

**Additional file 2: Table S1.** Geographical distribution and cultivated years of four ginseng cultivars.

**Additional file 3: Table S2.** The content of nine monosaccharides in each sample.

**Additional file 4: Table S3.** Summary of the four ginseng cultivars, sequencing and mapping based on the reference genome of 'chunpoog'.

**Additional file 5: Table S4.** Correlation analysis of genes involved in polysaccharides biosynthesis and polysaccharides content.

## Data Availability

The RNA-seq data has been submitted to NCBI SRA: PRJNA762437 and PRJNA779557.

## Abbreviations

PGM: Phosphoglucomutase
GPI: Glucose-6-phosphate isomerase
UGP2: UTP--glucose-1-phosphate uridylyltransferase
ScrK: Fructokinase
GMPP: Mannose-1-phosphate guanylyltransferase
PMM: Phosphomannomutase
GALE: UDP-glucose 4-epimerase
SacA: Beta-fructofuranosidase
SUS: Sucrose synthase
Ara: Arabinose
Rha: Rhamnose
Gal: Galactose
Xyl: Xylose
Xyl: Glucose
HPLC: High Performance Liquid Chromatography
PC: Principal components
DEGs: Differentially expressed genes
KEGG: Kyoto Encyclopedia of Genes and Genomes
GO: Gene ontology
D-fructose-6p: D-fructose-6phosphate
Man: Mannose
GlcA: Glc-acid
GalA: Gal-acid
Fuc: Fucose
UGP2: UTP-glucose-1-phosphate uridylyltransferas
PGM: Phosphoglucomutase
SUS: Sucrose synthase
GALE: UDP-glucose 4-epimerase
MPI: Mannose-6-phosphate isomerase
GMDS: GDP-mannose 4,6-dehydratase
UXS1: UDP-glucuronate decarboxylase
UXE: UDP-arabinose 4-epimerase
UGDH: UDP-glucose 6-dehydrogenase
GMPP: Guanylyltransferase
TSTA3: GDP-L-fucose synthase
GAE: UDP-glucuronate 4-epimerase
PMM: Phosphomannomutase
GPI: Glucose-6-phosphate isomerase
WGCNA: Weighted Gene Co-Expression Network Analysis
qRT-PCR: Quantitative real-time PCR
TFs: Transcription factors
