# Supplementary material for: Transcriptome reveals insights into biosynthesis of ginseng polysaccharides
Source: BMC Plant Biol. 2022 Dec 19;22:594. doi: 10.1186/s12870-022-03995-x (PMC9761977; doi:10.1186/s12870-022-03995-x)
Supplement: Supplementary file 1 — Additional file 1: Fig. S1. The heatmap of Pearson correlation coefficients (PCC) among biological repeats of each accession. Red represents the high correlation; blue represents the low correlation. R means root; S means stem; L means leaf. GL means GAOLI ginseng; CM means COMMON; SZ means SHIZHU ginseng; BT means BIANTIAO ginseng (BT). Fig. S2. The number of differentially expressed genes (DEGs) in each comparison group of different cultivars in the same tissue. GL means GAOLI ginseng; CM means COMMON; SZ means SHIZHU ginseng; BT means BIANTIAO ginseng (BT). Fig. S3. Gene Ontology (GO) enrichment analysis was performed for all DEGs from root in each comparison group. The bottom x-axis indicates represents the enrichment ratio of DEG (sample number/background number), and the y-axis represents each detailed classification of GO. Fig. S4. Gene Ontology (GO) enrichment analysis was performed for all DEGs from stem in each comparison group. The bottom x-axis indicates represents the enrichment ratio of DEG (sample number/background number), and the y-axis represents each detailed classification of GO. Fig. S5. Gene Ontology (GO) enrichment analysis was performed for all DEGs from leaf in each comparison group. The bottom x-axis indicates represents the enrichment ratio of DEG (sample number/background number), and the y-axis represents each detailed classification of GO. Fig. S6. Validation of the RNA-seq results by qRT-PCR, Bars show means of Log2 TPM (counts per length of transcript sequence per million mapped fragments) value and Log2 qRT-PCR value of two and three biological replicates, respectively. R: root; S: stem; L: leaf. [file 12870_2022_3995_MOESM1_ESM.docx]

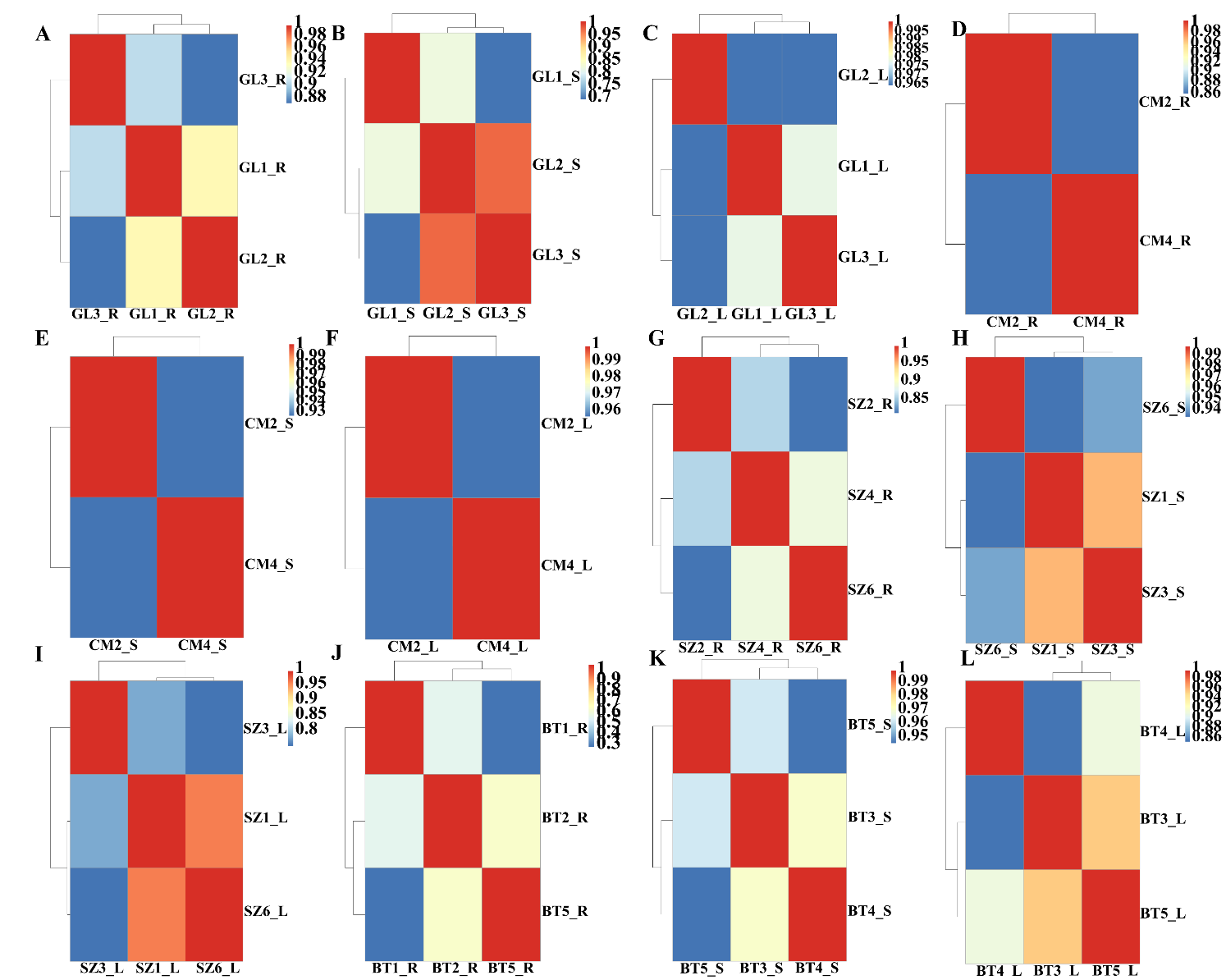
Fig. S1. The heatmap of Pearson correlation coefficients (PCC) among biological repeats of each accession. Red represents the high correlation; blue represents the low correlation. R means root; S means stem; L means leaf. GL means GAOLI ginseng; CM means COMMON; SZ means SHIZHU ginseng; BT means BIANTIAO ginseng (BT).


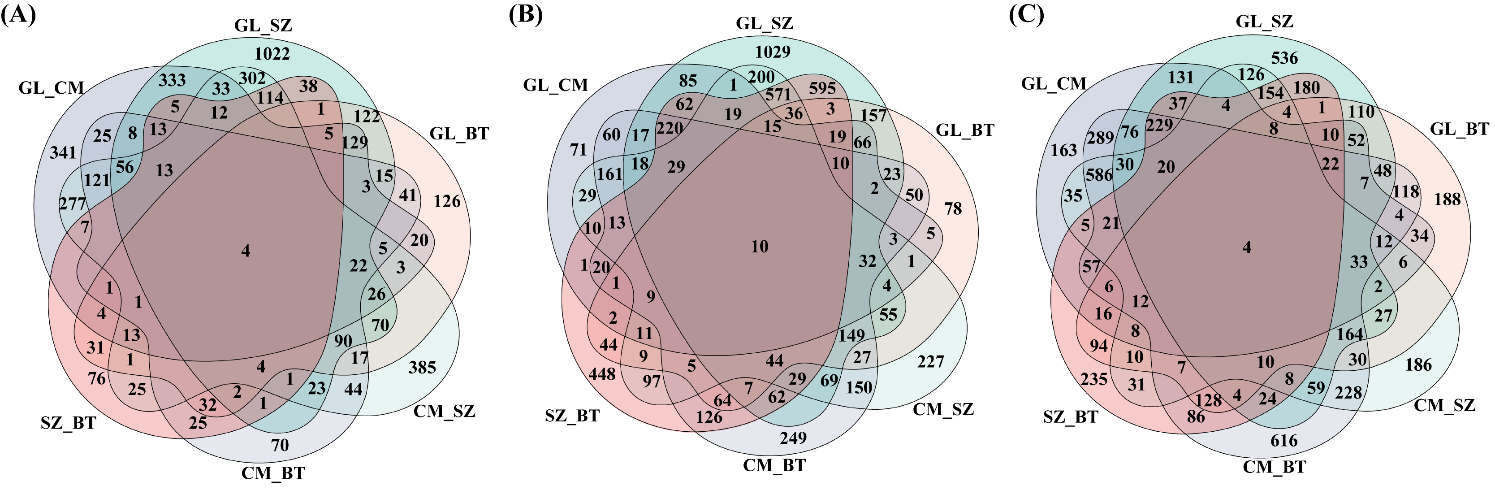


Fig. S2. The number of differentially expressed genes (DEGs) in each comparison group of different cultivars in the same tissue. GL means GAOLI ginseng; CM means COMMON; SZ means SHIZHU ginseng; BT means BIANTIAO ginseng (BT).


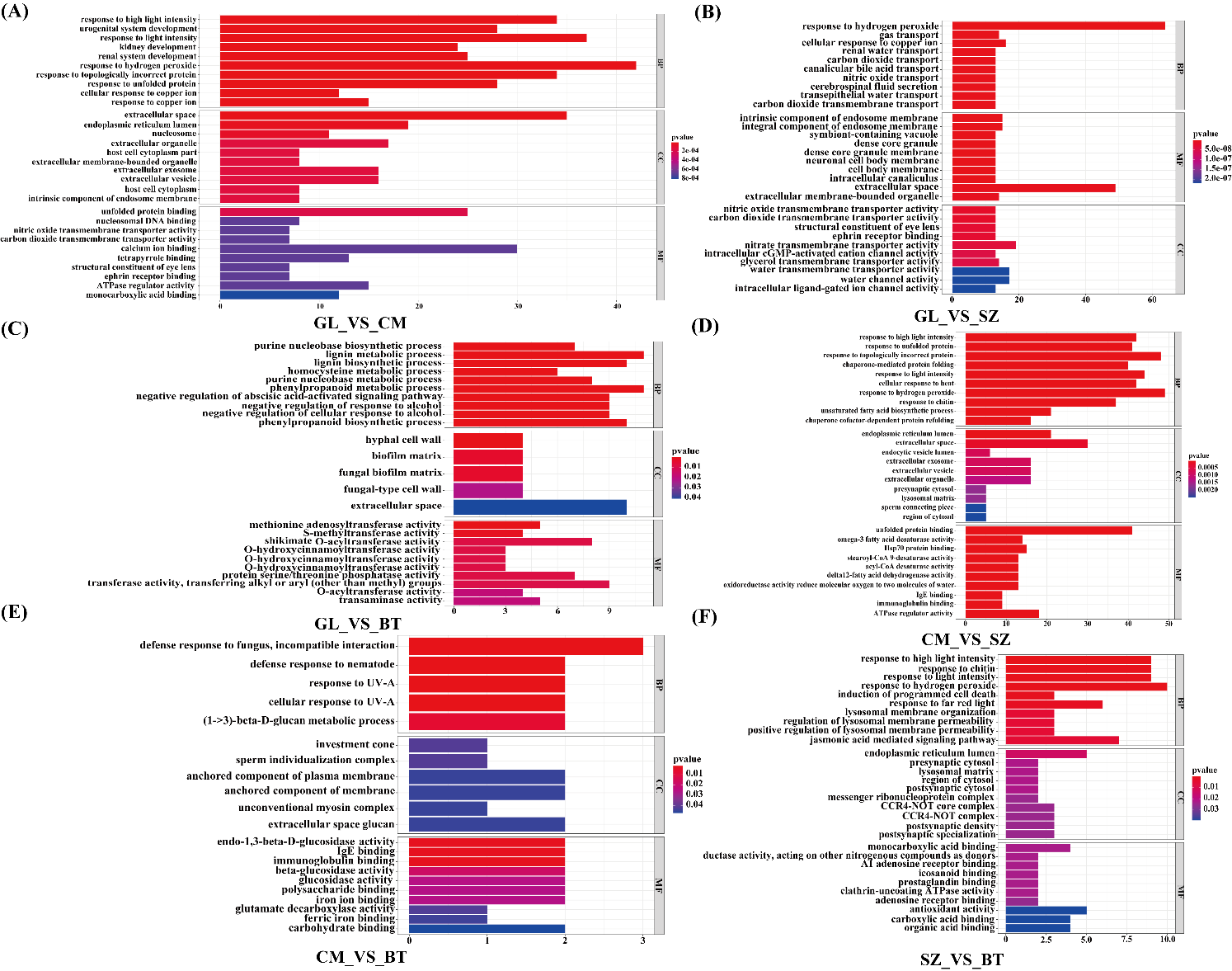


Fig. S3. Gene Ontology (GO) enrichment analysis was performed for all DEGs from root in each comparison group. The bottom x-axis indicates represents the enrichment ratio of DEG (sample number/background number), and the y-axis represents each detailed classification of GO.


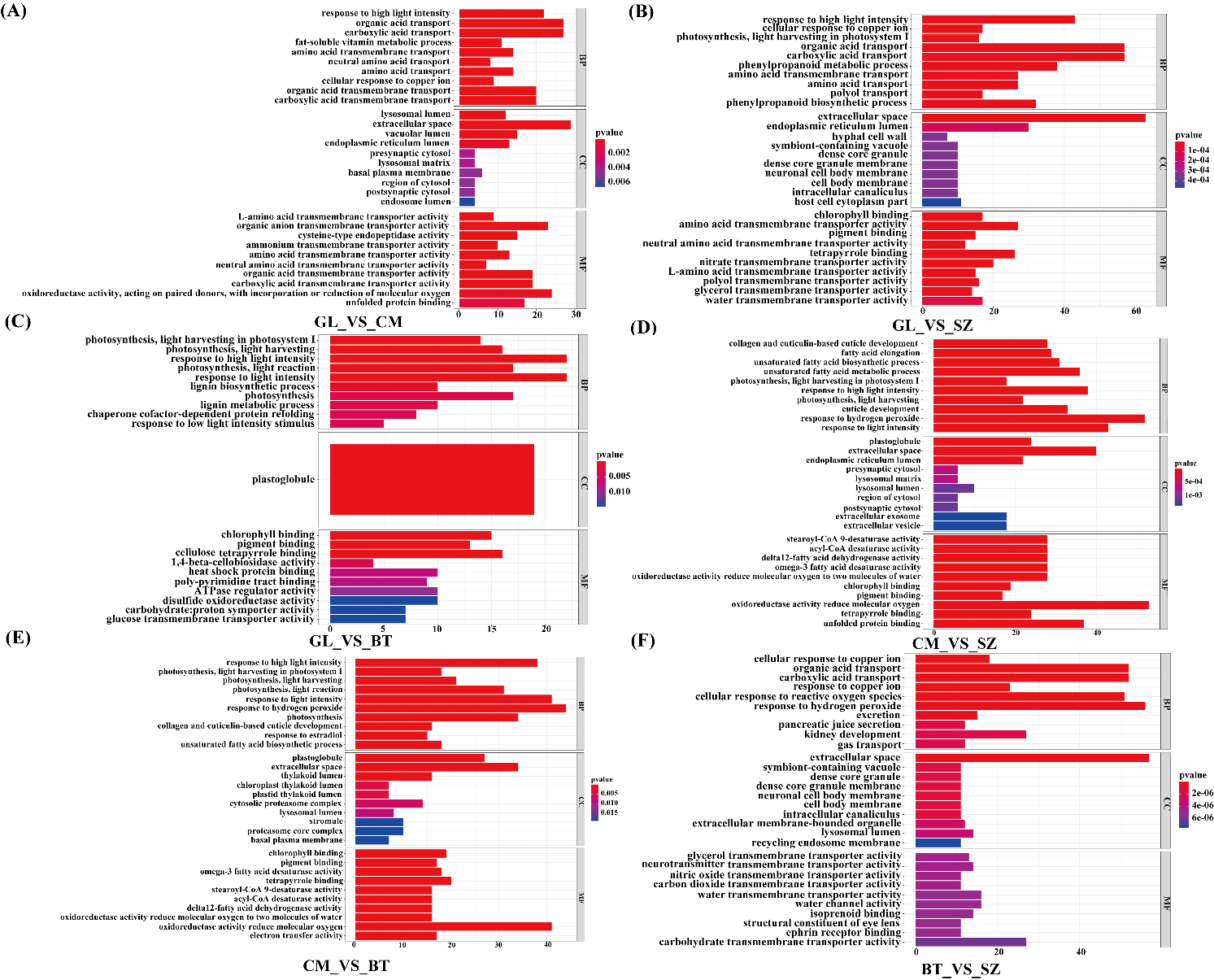


Fig. S4. Gene Ontology (GO) enrichment analysis was performed for all DEGs from stem in each comparison group. The bottom x-axis indicates represents the enrichment ratio of DEG (sample number/background number), and the y-axis represents each detailed classification of GO.


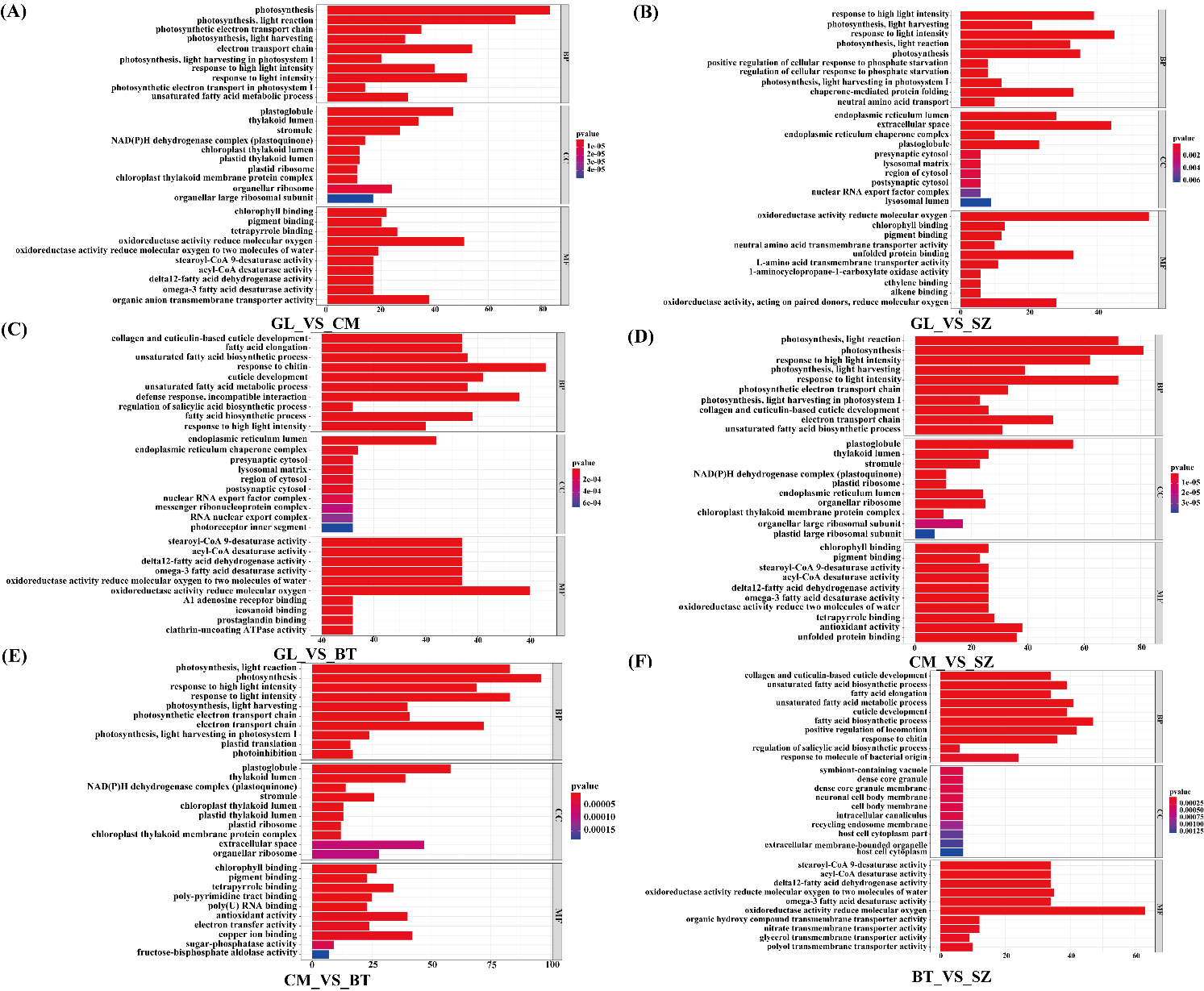


Fig. S5. Gene Ontology (GO) enrichment analysis was performed for all DEGs from leaf in each comparison group. The bottom x-axis indicates represents the enrichment ratio of DEG (sample number/background number), and the y-axis represents each detailed classification of GO.


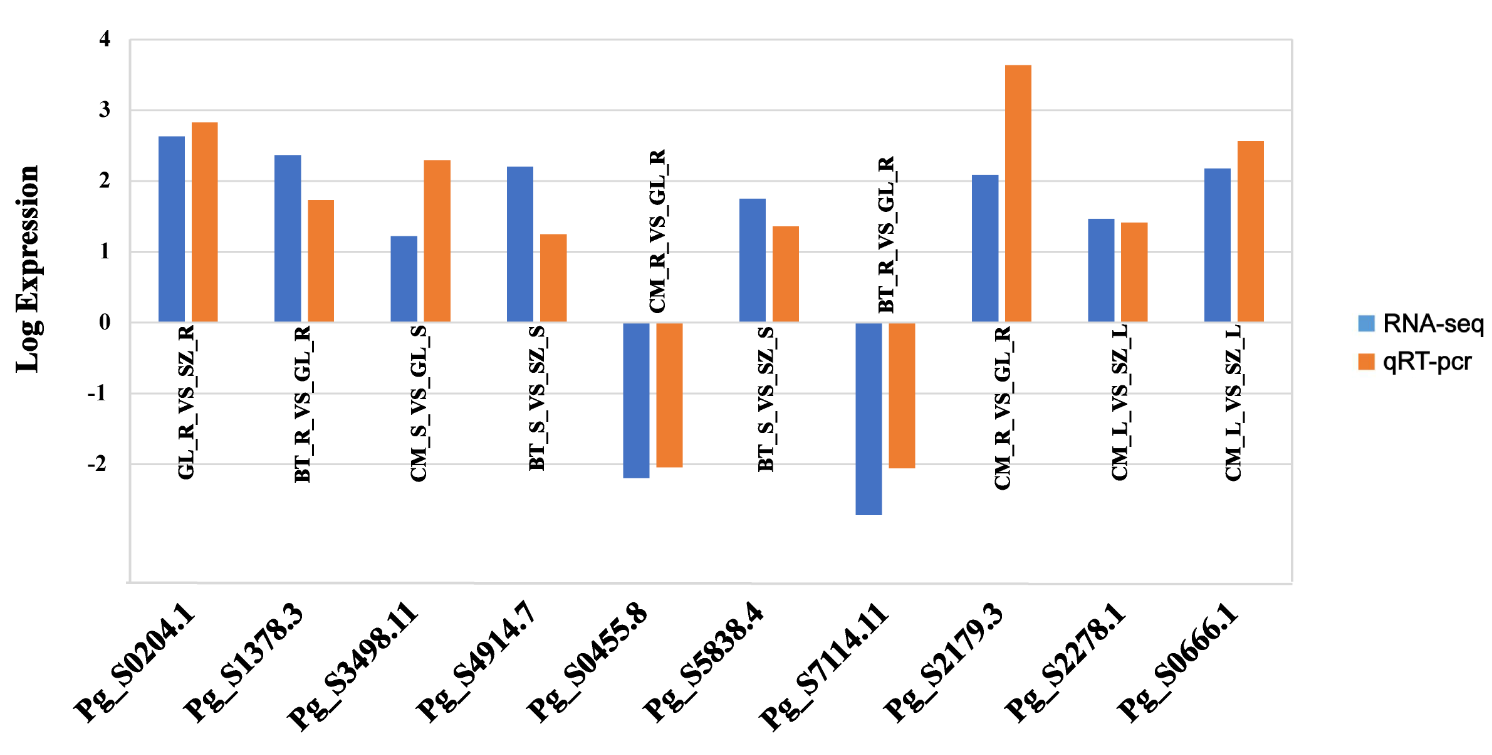


Fig. S6. Validation of the RNA-seq results by qRT-PCR, Bars show means of Log2 TPM (counts per length of transcript sequence per million mapped fragments) value and Log2 qRT-PCR value of two and three biological replicates, respectively. R: root; S: stem; L: leaf.
