# Supplementary material for: Transcriptome reveals insights into biosynthesis of ginseng polysaccharides
Source: BMC Plant Biol. 2022 Dec 19;22:594. doi: 10.1186/s12870-022-03995-x (PMC9761977; doi:10.1186/s12870-022-03995-x)
Supplement: Supplementary file 2 — Additional file 2: Table S1. Geographical distribution and cultivated years of four ginseng cultivars. [file 12870_2022_3995_MOESM2_ESM.docx]

Table S1. Geographical distribution and cultivated years of four ginseng cultivars.

| group | location | latitude | longitude | Altitude (m) | cultivars | cultivated time (year) |
| --- | --- | --- | --- | --- | --- | --- |
| GL | Korean Autonomous County of Changbai county | N41°25´12.06´´ | E128°12´02.84´´ | 712 | GAOLI ginseng | 6 |
| CM | Jingyu county | N42°23´19.32´´ | E126°48´46.81´´ | 547 | COMMON ginseng | 6 |
| SZ | Shizhuzi county | N40°46´07.53´´ | E125°50´47.98´´ | 163 | SHIZHU ginseng | 6 |
| BT | Taishang town | N41°17´54.67´´ | E125°50´47.98´´ | 355 | BIANTIAO ginseng | 6 |
