# Supplementary material for: Transcriptome reveals insights into biosynthesis of ginseng polysaccharides
Source: BMC Plant Biol. 2022 Dec 19;22:594. doi: 10.1186/s12870-022-03995-x (PMC9761977; doi:10.1186/s12870-022-03995-x)
Supplement: Supplementary file 3 — Additional file 3: Table S2. The content of nine monosaccharides in each sample. [file 12870_2022_3995_MOESM3_ESM.docx]

|  | root (mg/g) | | | | stem(mg/g) | | | | leaf (mg/g | | | |
| --- | --- | --- | --- | --- | --- | --- | --- | --- | --- | --- | --- | --- |
|  | GL | CM | SZ | BT | GL | CM | SZ | BT | GL | CM | SZ | BT |
| Man | 0.0290±0.0012a | 0.0358±0.0071a | 0.0268±0.0007a | 0.0307±0.0009a | 0.0277±0.0002a | 0.0267±0.0007a | 0.0271±0.0008a | 0.0276±0.0002a | 0.0339±0.0080a | 0.0270± 0.0010a | 0.0272± 0.0002a | 0.0288± 0.0007a |
| GlcA | 0.0587± 0.0042a | 0.0657±0.0128a | 0.0588±0.0080a | 0.0681±0.0063a | 0.0510±0.0002a | 0.0500±0.0001a | 0.0502±0.0002a | 0.0510±0.0007a | 0.0501± 0.0002a | 0.0500±0.0001a | 0.0503±0.0005a | 0.0524±0.0010a |
| Rha | 0.0382±0.0044a | 0.0477±0.0163a | 0.0340±0.0054a | 0.0455±0.0054a | 0.0292±0.0003a | 0.0283±0.0001a | 0.0281±0.0001a | 0.0288±0.0005a | 0.0292± 0.0006a | 0.0281± 0.0002a | 0.0287± 0.0007a | 0.0306± 0.0003a |
| GalA | 0.0389±0.0039a | 0.0497±0.0173a | 0.0365±0.0057a | 0.0488±0.0074a | 0.0304±0.0002a | 0.0293±0.0001b | 0.0294±0.0002b | 0.0294± 0.0001b | 0.0298± 0.0002a | 0.0310± 0.0020a | 0.0323± 0.0023a | 0.0304± 0.0006a |
| Glc | 15.6215±2.7941a | 17.2789±3.1008a | 4.2758±0.1621b | 15.0773±1.8104a | 1.5288±0.3546a | 1.1827±0.0284a | 1.5825±0.3432a | 1.3242±0.0548a | 1.3461±0.1003a | 1.4523±0.3000a | 1.1975±0.0181a | 1.4523±0.0570a |
| Gal | 0.0513±0.0023a | 0.0561±0.0038a | 0.0481±0.0043a | 0.0499±0.0052a | 0.0631±0.0027a | 0.0537±0.0065a | 0.0539±0.0034a | 0.0578±0.0024a | 0.0639±0.0064a | 0.0734± 0.0262a | 0.0722± 0.0031a | 0.0868± 0.0042a |
| Xyl | 0.0394±0.0023a | 0.0388±0.0007a | 0.0361±0.0011a | 0.0373±0.0028a | 0.0349±0.0004a | 0.0334± 0.0002a | 0.0346±0.0007a | 0.0346±0.0007a | 0.0337± 0.0003a | 0.0332± 0.000a | 0.0335± 0.0002a | 0.0346± 0.0004a |
| Ara | 0.0452±0.0012a | 0.0475±0.0002a | 0.0441±0.0017 | 0.0458±0.0022a | 0.0470±0.0010a | 0.0443±0.0017a | 0.0450±0.0010a | 0.0465± 0.0011a | 0.0485± 0.0022a | 0.0511± 0.0070a | 0.0511± 0.0014a | 0.0591± 0.0020a |
| Fuc | 0.0451±0.0002a | 0.0451±0.0001a | 0.0449±0.000a | 0.0452±0.0002a | 0.0448±0.000a | 0.0448±0.0001a | 0.0449±0.0001a | 0.0450±0.0001a | 0.0450± 0.0001a | 0.0450± 0.0003a | 0.0451± 0.0001a | 0.0451± 0.0001a |

Table S2. The content of nine monosaccharides in each sample.

Note: Different lowercase letters indicate significant differences among the four cultivars for the same monosaccharide in the roots, stems and leaves at the 0.05 level
