# Supplementary material for: Transcriptome reveals insights into biosynthesis of ginseng polysaccharides
Source: BMC Plant Biol. 2022 Dec 19;22:594. doi: 10.1186/s12870-022-03995-x (PMC9761977; doi:10.1186/s12870-022-03995-x)
Supplement: Supplementary file 4 — Additional file 4: Table S3. Summary of the four ginseng cultivars, sequencing and mapping based on the reference genome of 'chunpoog'. [file 12870_2022_3995_MOESM4_ESM.docx]

| Table S3. Summary of the four ginseng cultivars, sequencing and mapping based on the reference genome of 'chunpoog'**.** | | | | | | | |
| --- | --- | --- | --- | --- | --- | --- | --- |
| Sample ID | cultivars | Species | tissue | Clean Reads | Clean bases (G) | Q30 (%) | Map Rate (%) |
| GL1_R | GAOLI | *Panax ginseng* | root | 42490000.00 | 6.37 | 95.06 | 79.49 |
| GL2_R | GAOLI | *Panax ginseng* | root | 42600000.00 | 6.39 | 95.20 | 80.43 |
| GL3_R | GAOLI | *Panax ginseng* | root | 43070000.00 | 6.46 | 94.72 | 80.01 |
| CM2_R | COMMON | *Panax ginseng* | root | 41300000.00 | 6.19 | 93.33 | 77.57 |
| CM4_R | COMMON | *Panax ginseng* | root | 41290000.00 | 6.19 | 93.55 | 80.25 |
| SZ2_R | SHIZHU | *Panax ginseng* | root | 43030000.00 | 6.45 | 94.72 | 78.27 |
| SZ4_R | SHIZHU | *Panax ginseng* | root | 42610000.00 | 6.39 | 95.12 | 78.88 |
| SZ6_R | SHIZHU | *Panax ginseng* | root | 42780000.00 | 6.42 | 95.02 | 80.26 |
| BT1_R | BIANTIAO | *Panax ginseng* | root | 43010000.00 | 6.45 | 94.91 | 80.81 |
| BT2_R | BIANTIAO | *Panax ginseng* | root | 43040000.00 | 6.46 | 95.09 | 78.23 |
| BT5_R | BIANTIAO | *Panax ginseng* | root | 42840000.00 | 6.43 | 94.97 | 77.77 |
| GL1_J | GAOLI | *Panax ginseng* | stem | 44820000.00 | 6.72 | 93.09 | 79.03 |
| GL2_J | GAOLI | *Panax ginseng* | stem | 44750000.00 | 6.71 | 92.79 | 78.40 |
| GL3_J | GAOLI | *Panax ginseng* | stem | 44680000.00 | 6.70 | 92.59 | 78.53 |
| CM2_J | COMMON | *Panax ginseng* | stem | 43220000.00 | 6.48 | 92.78 | 78.53 |
| CM4_J | COMMON | *Panax ginseng* | stem | 43020000.00 | 6.45 | 92.66 | 78.92 |
| SZ1_J | SHIZHU | *Panax ginseng* | stem | 44420000.00 | 6.66 | 92.20 | 76.62 |
| SZ3_J | SHIZHU | *Panax ginseng* | stem | 44760000.00 | 6.71 | 93.18 | 78.43 |
| SZ6_J | SHIZHU | *Panax ginseng* | stem | 44780000.00 | 6.72 | 92.78 | 78.57 |
| BT3_J | BIANTIAO | *Panax ginseng* | stem | 44670000.00 | 6.70 | 93.25 | 77.54 |
| BT4_J | BIANTIAO | *Panax ginseng* | stem | 44820000.00 | 6.72 | 93.03 | 78.64 |
| BT5_J | BIANTIAO | *Panax ginseng* | stem | 44620000.00 | 6.69 | 93.41 | 78.41 |
| GL1_L | GAOLI | *Panax ginseng* | leaf | 43150000.00 | 6.47 | 92.56 | 76.83 |
| GL2_L | GAOLI | *Panax ginseng* | leaf | 43190000.00 | 6.48 | 92.58 | 76.88 |
| GL3_L | GAOLI | *Panax ginseng* | leaf | 42920000.00 | 6.44 | 92.68 | 76.68 |
| CM2_L | COMMON | *Panax ginseng* | leaf | 41990000.00 | 6.30 | 93.19 | 76.88 |
| CM4_L | COMMON | *Panax ginseng* | leaf | 41960000.00 | 6.29 | 92.97 | 78.20 |
| SZ1_L | SHIZHU | *Panax ginseng* | leaf | 43540000.00 | 6.53 | 93.66 | 80.52 |
| SZ3_L | SHIZHU | *Panax ginseng* | leaf | 43450000.00 | 6.52 | 92.95 | 78.19 |
| SZ6_L | SHIZHU | *Panax ginseng* | leaf | 43440000.00 | 6.52 | 92.91 | 78.35 |
| BT3_L | BIANTIAO | *Panax ginseng* | leaf | 42980000.00 | 6.45 | 92.81 | 77.42 |
| BT4_L | BIANTIAO | *Panax ginseng* | leaf | 43060000.00 | 6.46 | 92.83 | 76.87 |
| BT5_L | BIANTIAO | *Panax ginseng* | leaf | 43010000.00 | 6.45 | 92.95 | 77.33 |
