# Supplementary material for: Transcriptome reveals insights into biosynthesis of ginseng polysaccharides
Source: BMC Plant Biol. 2022 Dec 19;22:594. doi: 10.1186/s12870-022-03995-x (PMC9761977; doi:10.1186/s12870-022-03995-x)
Supplement: Supplementary file 5 — Additional file 5: Table S4. Correlation analysis of genes involved in polysaccharides biosynthesis and polysaccharides content. [file 12870_2022_3995_MOESM5_ESM.docx]

Table S4. Correlation analysis of genes involved in polysaccharides biosynthesis and polysaccharides content.

| Gene_id |  | Man | GlcA | Rha | GalA | Glc | Gal | Xyl | Ara | Fuc | Total polysaccharides |
| --- | --- | --- | --- | --- | --- | --- | --- | --- | --- | --- | --- |
| Pg_S2017.3  (PGM) | Cor  Sig.(2-tailed) | 0.510  0.003 | 0.438  0.014 | 0.525  0.002 |  |  |  | 0.402  0.025 | 0.387  0.031 |  |  |
| Pg_S4434.4  (HK) | Cor  Sig.(2-tailed) |  | -0.357  0.049 |  |  |  |  | -0.441  0.013 |  |  |  |
| Pg_S0635.5  (scrK) | Cor  Sig.(2-tailed) |  |  |  |  |  | 0.666  <0.001 |  | -0.702  <0.001 |  |  |
| Pg_S3013.7  (UXE) | Cor  Sig.(2-tailed) |  | 0.360  0.046 | 0.360  0.047 |  |  |  | 0.411  0.022 |  |  |  |
| Pg_S4588.10  (UXS1) | Cor  Sig.(2-tailed) |  | 0.453  0.010 | 0.415  0.020 |  |  |  | 0.494  0.005 |  |  |  |
| Pg_S5470.8  (SUS) | Cor  Sig.(2-tailed) |  |  |  |  | 0.420  0.019 |  |  |  |  | 0.414  0.021 |
| Pg_S7084.1  (sacA) | Cor  Sig.(2-tailed) |  | 0.360  0.047 | 0.423  0.018 |  | 0.363  0.045 | -0.402  0.025 | 0.420  0.019 |  |  | 0.392  0.029 |
| Pg_S5853.14  (UXS1) | Cor  Sig.(2-tailed) |  | 0.478  0.006 | 0.565  0.001 |  |  |  | 0.472  0.007 |  |  |  |
| Pg_S7836.3  (GMPP) | Cor  Sig.(2-tailed) |  | 0.547  0.001 | 0.576  0.001 | 0.507  0.004 | 0.689  <0.001 | -0.453  0.010 | 0.634  <0.001 |  |  | 0.686  <0.001 |
| Pg_S0917.35  (PGM) | Cor  Sig.(2-tailed) |  | 0.402  0.025 | 0.389  0.031 |  | 0.362  0.046 | -0.5558  <0.001 | 0.526  0.002 | -0.537  0.002 |  |  |
| Pg_S3346.1  (HK) | Cor  Sig.(2-tailed) |  |  |  |  | 0.423  0.018 | -0.398  0.027 |  |  |  | 0.407  0.023 |
| Pg_S1347.13  (SUS) | Cor  Sig.(2-tailed) |  | 0.409  0.023 | 0.440  0.013 | 0.449  0.011 | 0.428  0.016 |  | 0.545  0.002 |  |  | 0.410  0.022 |
| Pg_S5058.9  (UXS1) | Cor  Sig.(2-tailed) | 0.418  0.019 | 0.512  0.003 | 0.601  <0.001 |  |  |  | 0.419  0.019 |  |  | 0.360  0.046 |
| Pg_S0458.3  (PGM) | Cor  Sig.(2-tailed) |  |  |  |  |  | 0.613  <0.001 |  | 0.504  0.004 |  |  |
| Pg_S7036.4  (GPI) | Cor  Sig.(2-tailed) |  |  |  |  |  | -0.541  0.002 | 0.449  0.0111 | -0.517  0.003 | -0.453  0.010 |  |
| Pg_S0953.13  (GMPP) | Cor  Sig.(2-tailed) |  | 0.526  0.002 |  |  | 0.433  0.015 | -0.608  <0.001 | 0.570  0.001 | -0.460  0.009 |  | 0.421  0.009 |
| Pg_S1306.14  (scrK) | Cor  Sig.(2-tailed) |  |  |  |  |  | 0.380  0.035 |  | 0.513  0.003 |  |  |
| Pg_S1290.1  (GAE) | Cor  Sig.(2-tailed) |  |  |  |  |  | -0.467  0.008 | 0.397  0.027 | -0.526  0.002 |  |  |
| Pg_S4516.21  (PMM) | Cor  Sig.(2-tailed) |  | 0.480  0.006 | 0.383  0.033 |  |  | -0.419  0.019 | 0.491  0.005 |  |  |  |
| Pg_S2020.12  (UGDH) | Cor  Sig.(2-tailed) | -0.409  0.023 | -0.590  <0.001 | -0.455  0.010 |  | -0.389  0.030 |  | -0.586  0.001 |  |  | -0.394  0.028 |
| Pg_S4342.7  (UXS1) | Cor  Sig.(2-tailed) | 0.446  0.012 | 0.514  0.003 | 0.465  0.008 |  |  | -0.417  0.019 | 0.496  0.005 |  |  |  |
| Pg_S1124.2  (UGP2) | Cor  Sig.(2-tailed) |  | 0.535  0.002 | 0.509  0.003 | 0.382  0.034 | 0.474  0.007 | -0.462  0.009 | 0.692  <0.001 | -0.394  0.028 |  | 0.442  0.013 |
| Pg_S0897.14  (GALE) | Cor  Sig.(2-tailed) |  |  |  |  |  |  |  |  | -0.403  0.025 | -0.375  0.038 |
| Pg_S0808.14  (UGDH) | Cor  Sig.(2-tailed) |  |  |  |  | -0.469  0.008 |  |  |  | -0.396  0.028 | -0.489  0.005 |
| Pg_S1501.44  (TSTA3) | Cor  Sig.(2-tailed) |  |  |  |  |  | -0.614  0<001 | 0.454  0.010 | -0.614  <0.001 |  |  |
| Pg_S1886.12  (GMPP) | Cor  Sig.(2-tailed) |  | 0.375  0.038 |  |  | 0.432  0.015 | -0.385  0.032 |  |  |  | 0.451  0.011 |
| Pg_S4425.1  (UXE) | Cor  Sig.(2-tailed) |  |  |  |  | 0.383  0.033 |  |  |  |  | 0.403  0.025 |
| Pg_S5644.4  (MPI) | Cor  Sig.(2-tailed) |  |  |  | -0.360  0.046 |  |  |  | 0.357  0.048 |  |  |
| Pg_S8336.3  (scrK) | Cor  Sig.(2-tailed) |  |  | -0.396  0.028 | -0.570  0.001 | -0.482  0.006 |  | -0.363  0.045 |  |  | -0.505  0.004 |
| Pg_S1563.8  (UXS1) | Cor  Sig.(2-tailed) | 0.449  0.011 | 0.639  <0.001 | 0.514  0.003 |  | 0.687  <0.001 | -0.520  0.003 | 0.615  <0.001 |  |  | 0.676  <0.001 |
| Pg_S2035.27  (UGP2) | Cor  Sig.(2-tailed) |  | 0.482  0.006 | 0.441  0.013 | 0.606  <0.001 | 0.555  0.001 | -0.595  0.001 | 0.529  0.002 |  |  | 0.548  0.001 |
| Pg_S4929.12  (HK) | Cor  Sig.(2-tailed) | -0.434  0.015 | -0.572  0.001 | -0.473  0.007 | -0.421  0.018 |  |  | -0.425  0.017 |  |  | -0.361  0.046 |
| Pg_S4083.8  (SUS) | Cor  Sig.(2-tailed) |  |  |  |  | -0.361  0.046 | 0.421  0.018 |  |  |  | -0.370  0.040 |
| Pg_S1495.1  (scrK) | Cor  Sig.(2-tailed) |  |  |  |  | 0.388  0.031 | -0.494  0.005 |  |  |  | 0.382  0.034 |
| Pg_S0061.8  (sacA) | Cor  Sig.(2-tailed) |  |  |  | -0.566  0.001 | -0.473  0.007 | 0.441  0.013 | -0.479  0.006 | 0.369  0.038 |  | -0.461  0.009 |
| Pg_S0758.5  (UXS1) | Cor  Sig.(2-tailed) |  | 0.417  0.020 |  |  | 0.381  0.035 | -0.506  0.004 | 0.371  0.040 |  |  | 0.367  0.042 |
| Pg_S0496.3  (UXS1) | Cor  Sig.(2-tailed) |  |  |  |  |  |  |  | 0.388  0.031 |  |  |
| Pg_S0219.46  (UGDH) | Cor  Sig.(2-tailed) |  |  |  |  |  |  |  | -0.437  0.014 |  |  |
| Pg_S3876.17  (sacA) | Cor  Sig.(2-tailed) |  | -0.437  0.014 |  | -0.416  0.020 | -0.531  0.002 | 0.532  0.002 | -0.538  0.002 | 0.432  0.015 |  | -0.525  0.002 |
| Pg_S5989.1  (GALE) | Cor  Sig.(2-tailed) |  |  |  |  | -0.402  0.025 | 0.508  0.004 |  | 0.398  0.027 |  | -0.400  0.026 |
| Pg_S4164.13  (UGP2) | Cor  Sig.(2-tailed) |  |  |  |  | 0.376  0.037 | -0.444  0.012 |  |  |  | 0.357  0.049 |
| Pg_S1171.27  (UXE) | Cor  Sig.(2-tailed) |  |  |  |  |  | -0.431  0.015 |  |  | 0.367  0.042 |  |
| Pg_S2316.15  (UXS1) | Cor  Sig.(2-tailed) |  | 0.602  <0.001 | 0.431  0.016 | 0.419  0.019 | 0.716  <0.001 | -0.479  0.006 | 0.643  <0.001 |  |  | 0.711  <0.001 |
| Pg_S1242.6  (UGDH) | Cor  Sig.(2-tailed) |  |  |  |  | -0.454  0.010 |  |  |  | -0.557  0.001 | -0.476  0.007 |
| Pg_S0588.13  (scrK) | Cor  Sig.(2-tailed) |  |  |  | 0.545  0.002 | 0.390  0.030 |  |  |  |  | 0.414  0.021 |
| Pg_S0234.21  (HK) | Cor  Sig.(2-tailed) |  |  |  | -0.471  0.007 |  |  |  |  |  |  |
| Pg_S8075.1  (GPI) | Cor  Sig.(2-tailed) |  |  |  |  |  | 0.661  <0.001 |  | 0.670  <0.001 |  |  |
| Pg_S2021.6  (GAE) | Cor  Sig.(2-tailed) |  |  |  | 0.431  0.015 | 0.400  0.026 | -0.433  0.015 |  |  |  | 0.422  0.018 |
| Pg_S1915.25  (GMPP) | Cor  Sig.(2-tailed) |  | 0.482  0.006 | 0.375  0.038 | 0.587  0.001 | 0.630  <0.001 |  | 0.540  0.002 |  |  | 0.647  <0.001 |
| Pg_S3604.8  (GALE) | Cor  Sig.(2-tailed) |  |  |  |  | -0.437  0.014 | 0.368  0.042 |  |  |  | -0.468  0.008 |
| Pg_S3302.8  (UGP2) | Cor  Sig.(2-tailed) | 0.415  0.020 | 0.462  0.009 | 0.472  0.007 |  | 0.380  0.035 |  | 0.490  0.005 |  |  | 0.388  0.031 |
| Pg_S3338.6  (GMPP) | Cor  Sig.(2-tailed) |  |  |  |  |  | 0.490  0.005 |  | 0.475  0.007 |  |  |
| Pg_S5422.1  (SUS) | Cor  Sig.(2-tailed) | 0.394  0.028 | 0.649  <0.001 | 0.434  0.015 |  | 0.619  <0.001 | -0.509  0.003 | 0.798  <0.001 |  |  | 0.585  0.001 |
| Pg_S5155.1  (scrK) | Cor  Sig.(2-tailed) |  | 0.479  0.006 | 0.507  0.004 | 0.376  0.037 | 0.501  0.004 |  | 0.649  <0.001 |  |  | 0.483  0.006 |
| Pg_S5279.7  (PGM) | Cor  Sig.(2-tailed) |  |  | 0.380  0.035 |  |  |  | 0.440  0.013 | -0.376  0.037 |  |  |
| Pg_S0167.13  (GPI) | Cor  Sig.(2-tailed) |  |  |  |  |  | -0.525  0.002 | 0.446  0.012 | -0.572  0.001 | -0.458  0.010 |  |
| Pg_S1147.6  (PGM) | Cor  Sig.(2-tailed) | 0.383  0.033 | 0.378  0.036 | 0.457  0.010 | 0.645  <0.001 | 0.575  0.001 |  | 0.439  0.014 |  |  | 0.590  <0.001 |
| Pg_S2241.31  (scrK) | Cor  Sig.(2-tailed) |  | 0.583  0.001 | 0.478  0.007 | 0.459  0.009 | 0.571  0.001 | -0.502  0.004 | 0.639  <0.001 |  |  | 0.545  0.002 |
| Pg_S1305.19  (UGDH) | Cor  Sig.(2-tailed) |  | 0.414  0.021 |  |  |  |  |  |  |  |  |
| Pg_S0113.8  (GAE) | Cor  Sig.(2-tailed) |  |  |  |  |  | 0.407  0.023 |  |  |  |  |
| Pg_S1064.5  (GPI) | Cor  Sig.(2-tailed) |  |  |  | 0.513  0.003 |  |  |  |  |  |  |
| Pg_S3830.6  (SUS) | Cor  Sig.(2-tailed) |  |  |  |  |  |  |  | -0.359  0.047 |  |  |
| Pg_S5794.4  (SUS) | Cor  Sig.(2-tailed) | 0.387  0.032 | 0.551  0.001 | 0.601  <0.001 |  | 0.511  0.003 | -0.451  0.011 | 0.679  <0.001 |  |  | 0.485  0.006 |
| Pg_S0455.9  (GMPP) | Cor  Sig.(2-tailed) |  |  |  |  |  |  |  | -0.387  0.031 |  |  |
| Pg_S3153.2  (scrK) | Cor  Sig.(2-tailed) |  | 0.555  0.001 | 0.408  0.023 |  | 0.538  0.002 | -0.714  <0.001 | 0.606  <0.001 | -0.612  <0.001 |  | 0.507  0.004 |
| Pg_S4460.5  (GMDS) | Cor  Sig.(2-tailed) |  |  |  |  |  | -0.397  0.027 |  |  |  |  |

Note: Cor, Coefficient of correlation; Sig, P valve.
